# A journey from molecule to physiology and *in silico* tools for drug discovery targeting the transient receptor potential vanilloid type 1 (TRPV1) channel

**DOI:** 10.3389/fphar.2023.1251061

**Published:** 2024-01-24

**Authors:** Cesar A. Amaya-Rodriguez, Karina Carvajal-Zamorano, Daniel Bustos, Melissa Alegría-Arcos, Karen Castillo

**Affiliations:** ^1^ Centro Interdisciplinario de Neurociencia de Valparaíso, Facultad de Ciencias, Universidad de Valparaíso, Valparaíso, Chile; ^2^ Departamento de Fisiología y Comportamiento Animal, Facultad de Ciencias Naturales, Exactas y Tecnología, Universidad de Panamá, Ciudad de Panamá, Panamá; ^3^ Centro de Investigación de Estudios Avanzados del Maule (CIEAM), Vicerrectoría de Investigación y Postgrado Universidad Católica del Maule, Talca, Chile; ^4^ Laboratorio de Bioinformática y Química Computacional, Departamento de Medicina Traslacional, Facultad de Medicina, Universidad Católica del Maule, Talca, Chile; ^5^ Núcleo de Investigación en Data Science, Facultad de Ingeniería y Negocios, Universidad de las Américas, Santiago, Chile

**Keywords:** temperature, TRP channels, drug discovery, pain, structure, transient receptor potential vanilloid type 1 (TRPV1) channel

## Abstract

The heat and capsaicin receptor TRPV1 channel is widely expressed in nerve terminals of dorsal root ganglia (DRGs) and trigeminal ganglia innervating the body and face, respectively, as well as in other tissues and organs including central nervous system. The TRPV1 channel is a versatile receptor that detects harmful heat, pain, and various internal and external ligands. Hence, it operates as a polymodal sensory channel. Many pathological conditions including neuroinflammation, cancer, psychiatric disorders, and pathological pain, are linked to the abnormal functioning of the TRPV1 in peripheral tissues. Intense biomedical research is underway to discover compounds that can modulate the channel and provide pain relief. The molecular mechanisms underlying temperature sensing remain largely unknown, although they are closely linked to pain transduction. Prolonged exposure to capsaicin generates analgesia, hence numerous capsaicin analogs have been developed to discover efficient analgesics for pain relief. The emergence of *in silico* tools offered significant techniques for molecular modeling and machine learning algorithms to indentify druggable sites in the channel and for repositioning of current drugs aimed at TRPV1. Here we recapitulate the physiological and pathophysiological functions of the TRPV1 channel, including structural models obtained through cryo-EM, pharmacological compounds tested on TRPV1, and the *in silico* tools for drug discovery and repositioning.

## 1 Introduction

Throughout evolution, different groups of organisms have developed highly conserved proteins. These proteins enable them to perceive and integrate fluctuations in environmental cues, including temperature. Temperature is a pervasive physical signal that varies in time and space, significantly affecting the metabolism, physiology, and behavior of organisms. Animals and other organisms have developed complex proteins that allow them to perceive changes in temperature and respond physiologically to adapt, survive, and reproduce. This process is crucial for their survival and evolution. The perception of temperature changes and other stimuli is facilitated by the expression of specialized receptors in the cell membrane. These receptors aid in the perception, interpretation, and differentiation of varying temperature ranges, allowing organisms to appropriately respond and adapt to external conditions. However, sensing errors can have detrimental and potentially fatal consequences for survival.

Temperature sensing is made possible through the expression of a subset of channel receptors that belong to the transient receptor potential (TRP) ion channel superfamily. These receptors are known as thermoTRP channels. The origin of the name TRP comes from the discoveries made by Cosens and Manning, (1969), with a mutant strain of *Drosophila melanogaster* that shows an abnormal transient response to light instead of a sustained receptor potential during light exposure that is normally observed in the wild phenotype, then the mutant was named trp (transient receptor potential). In 1989 the gene responsible for this trp phenotype was cloned in *D. melanogaster* and was suggested that may function as a channel or receptor (Montell and Rubin, 1989). Homologues of these channels were subsequently identified in several taxa, and currently 28 members of the TRP channel superfamily have been identified, grouped into eight subfamilies: TRPC (“canonical”), TRPA (“ankyrin”), TRPV (“vanilloid”), TRPM (“melastatin”), TRPS (“soromelastatin”), TRPML (“mucolipin”), TRPP (“polycystin”) and TRPN (“Non-mecano-potential or NOMP-C”) (Himmel et al., 2021). Most TRP channels function as polymodal receptors that detect and transduce physical and chemical signals such as pressure, pH, temperature, lipids, toxins, chemical ligands, and other stimuli. Functional TRP channels are tetramers formed by subunits with a topology of six transmembrane segments with the pore domain between the S5-S6 segments, and the N- and C-terminal domains facing intracellularly (Gees et al., 2012). A subset of TRP channels belonging to TRPV (1–4), TRPM (2, 3, 4, 5, 8), TRPA1 and TRPC5, are thermally gated and respond to temperatures ranging from noxious cold (<15°C) to noxious heat (>42°C) and are classified as “thermoTRPs” (Zimmermann et al., 2011; Ferrandiz-Huertas et al., 2014; Song et al., 2016). The TRPV1 known for its involvement in nociception and pain, is a highly-researched member of thermoTRP channels. Biomedical studies aim to identify suitable molecules for pain relief therapeutic intervention, with particular focus on this ion channel.

### 1.1 Transient receptor potential vanilloid type 1 channel

**FIGURE 1:**
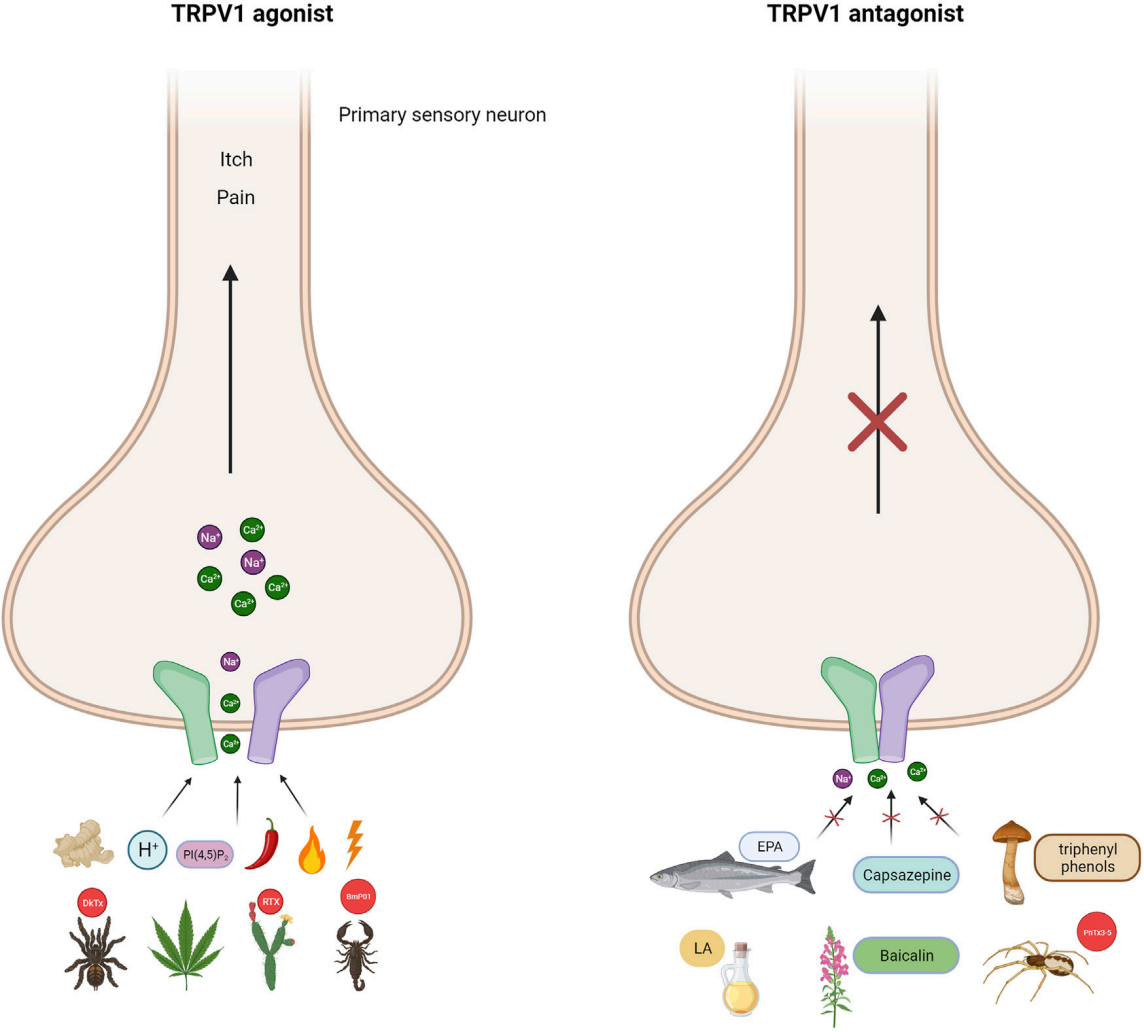
TRPV1 channel as polymodal receptor. TRPV1 channels in mammals can be activated by a range of ligands including heat above 42°C, capsaicin (the compound responsible for spicy in peppers), voltage, endogenous stimuli such as protons, lipids PI(4,5)P_2_, ginger (gingerol), *Cannabis sativa* (endocannabinoids), and plant and animal toxins. Indeed, resiniferatoxin (RTX) from the native Moroccan species *Euphorbia resinifera*, the native Nigerian species *Einhorbia possonii*, DkTx peptide toxins from the terrestrial tiger tarantula (*Ornithoctonus huwena*), and BmP01 from the Chinese scorpion (*Mesobuthus martensii*). This activation makes the flow of cations more permeable, causing pain and itching. Antagonists of the TRPV1 channel on the other hand, cause the channel to close, blocking its activity. Capsazepine is a synthetic compound structurally similar to capsaicin and is a classic antagonist of TRPV1 channels. Fascinating antagonists of natural origin have also been discovered, such as eicosapentaenoic acid (EPA), derived from fish, and linoleic acid (LA), mainly present in oils, both of which are fatty acids. The group of flavonoids includes baicalin from the plant *Scutellaria baicalensis* and triprenyl phenols (albaconol, grifolina, neogrifolina) from the fungal genus *Albatrellus spp*. Additionally, the peptide toxin PnTx3-5 from the banana spider *Phoneutria nigrivente* is also a member of this group. The broad range of TRPV1 modulators allows for the development of new analgesics which can act as either TRPV1 agonists or antagonists (Created with BioRender.com)

**FIGURE 2:**
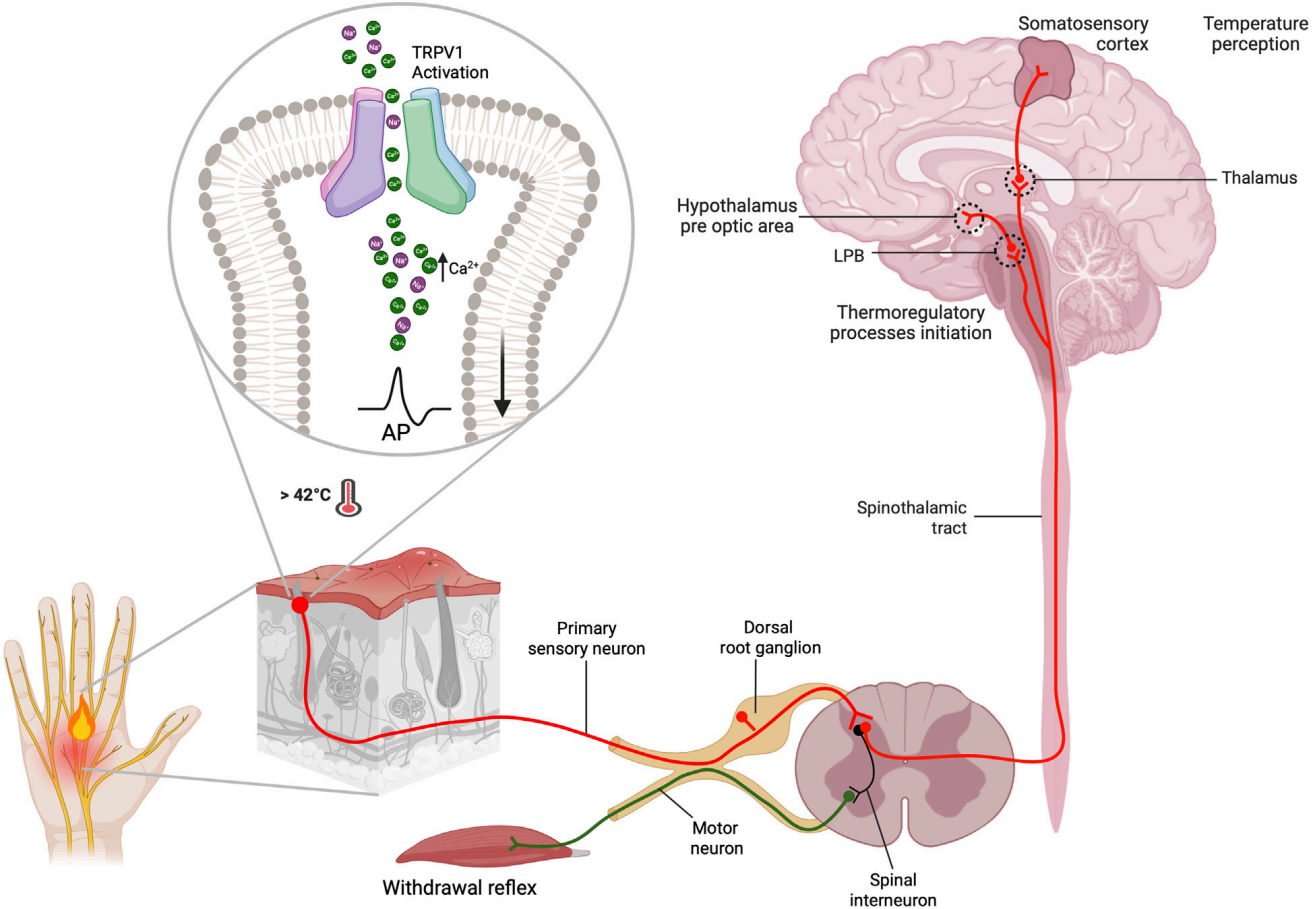
Thermosensation and thermoregulation processes occur from the periphery to the central nervous system. TRPV1 channels are mainly located in the primary afferent neurons (C and Aδ fibers) of the somatosensory system. They function as the primary integrator of noxious stimuli, leading to thermosensation and pain perception. After being activated by a noxious stimulus, such as a temperature above 42°C, nociceptors allow Ca^2+^ influx. This leads to local membrane depolarization by opening voltage-dependent sodium channels, which consequently generates afferent action potentials. These action potentials allow information to propagate to the central nervous system. The action potentials generated in the periphery travel to the dorsal horn. There, they form two types of synapses: one with motoneurons via spinal interneurons to give rise to the withdrawal reflex, and another with sensory neurons of the spinothalamic tract. The information transmitted at this synapse gives rise to two important processes: thermosensation and thermoregulation. The perception of temperature originates in the primary somatosensory cortex after the information is transmitted from the thalamus through thalamocortical radiations, where. Additionally, the information is transmitted to the hypothalamus, specifically to the preoptic area (POAI, via lateral parabrachial neurons (LPB), to initiate thermoregulatory processes (Created with BioRender.com)

The TRPV1 channel, initially named the capsaicin and heat receptor (VR1) (Montell et al., 2002), was the first thermoTRP channel to be identified and characterized from the dorsal root ganglion (DRG) in 1997. It can be activated by temperature >42°C, protons, and the pungent compound found in chili peppers called capsaicin (Caterina et al., 1997). TRPV1 channels are non-selective cationic channels that preferentially allow the passage of Ca^2+^ ions. They can be modulated by a multitude of stimuli, including changes in voltage or pH, as well as the presence of lipids, vanilloids, phyto- and endocannabinoids, PI (4,5)P_2_, a variety of chemical compounds and pharmacological agents. These channels have the potential to be useful in biomedical applications (Figure 1) (Szallasi et al., 2007). At the sensory nerve endings, the activation of TRPV1 channels by temperature causes an influx Ca^2+^ ions and membrane depolarization. This initiates the generation of action potentials that synapse at the dorsal root ganglion and propagate to the CNS, generating thermosensation and thermoregulatory processes, as well as the perception of pain (Julius, 2013). The perception of pain involves numerous signaling pathways and relays within the central nervous system, leading to complex processing. ThermoTRP channels expressed in free nerve endings play a critical role in various physiological and pathophysiological pain types, including but not limited to neuropathic, mechanical, inflammatory, dental, migraine, and visceral pain (Mickle et al., 2016). Numerous inflammatory agents, such as protons, prostaglandins, bradykinin, reactive oxygen species, interleukins, histamines, and others, have the capability to modulate and sensitize thermoTRP channels. As a result, these ion channels act as the molecular basis for noxious peripheral, physical, thermal and chemical stimuli. Electrical signal conversion from the detected stimuli commence in the peripheral tissues upon the activation of thermoTRP channels. The Ca2+ influx depolarize C or Aδ fibers increasing action potential firing rate. The electric signal travels to the dorsal root of the spinal cord, where it can produce the stimulation of a motor neuron, resulting in a rapid withdrawal reflex to a noxious stimulus. Alternatively, it can ascend through pathways of the brainstem, synapse in the thalamus, and ultimately reach the somatosensory cortex or preoptic area. These regions are responsible for the perception of temperature and thermoregulation, respectively (Figure 2).

The sustained exposure of TRPV1 to agonists, such as capsaicin, causes its desensitization, stopping its function, which produces analgesia, thus the search for capsaicin analogs for pain relief has been the focus of intense biomedical research. The associated mechanisms for TRPV1 desensitization appear to occur by pore closure, or by tachyphylaxis (Touska et al., 2011; Luo et al., 2019; Tian et al., 2019). In the former a reduction in pore diameter prevents ion permeation through the channel, whereas in the latter, channel internalization occurs with recycling to the plasma membrane with a temporal recovery depending on stimulus intensity. It has been suggested that long-term desensitization of TRPV1 may involve its removal from the plasma membrane by a clathrin-independent endocytic mechanism (Sanz-Salvador et al., 2012). The discovery of the ion channels acting as temperature receptors, and touch receptors, ion channels leads to David Julius and Ardem Patapoutian being awarded the Nobel Prize in Physiology or Medicine in 2021 (Latorre and Díaz-Franulic, 2022).

TRPV1 channels are expressed in excitable and non-excitable cells and tissues, where is associated with several physiological and pathophysiological conditions, including nociception and pain. The expression of TRPV1 is found throughout the peripheral nervous system and is predominant in small myelinated (Aδ) and unmyelinated (C) fibers from sensory ganglia of the dorsal root ganglion (DRG) in the spinal cord, the trigeminal ganglia, and nodose ganglia neurons (Caterina et al., 1997; Hwang and Valtschanoff, 2003). A role in memory and learning in the CNS has also been proposed. Although there is no certainty about the precise location of TRPV1 at central synapses, it is known that the receptor can modulate both neurotransmitter release at presynaptic terminals, as well as the synaptic efficacy in postsynaptic compartments (Matta et al., 2010; Meza et al., 2022). In fact, it has been observed that TRPV1 activity suppresses the excitatory transmission in the dentate gyrus of the hippocampus, and that its synaptic activation induces a form of long-term depression, mediated by endogenous anandamide (Chávez et al., 2010). TRPV1 is also expressed in corneal afferent neurons and in the cortical areas, and in the lamina I and II of the dorsal horn of the spinal cord (Fernandes et al., 2012; Mickle et al., 2015; Morales-Lázaro and Rosenbaum, 2015). Its subcellular expression has been also been found at dendritic spines and synaptic vesicles (Ho et al., 2012), as well as in various neurons of the brain, including all cortical areas, hippocampus, and dentate gyrus, fimbrial, dorsal and lateral septal nuclei, central amygdala, medial and lateral habenula, mampillary and interpeduncular nuclei, stria terminalis, suprachiasmatic nucleus, inferior olive, and others (Mezey et al., 2000), human sympathetic ganglia (Kokubun et al., 2015), smooth muscle cells (Kark et al., 2008), endothelial cells (Golech et al., 2004), liver (Li et al., 2012), colon (Matsumoto et al., 2009), pancreas (Akiba et al., 2004), lung (Johansen et al., 2006), bladder and male genital tract (Stein et al., 2004), uterus (Contassot et al., 2004), stomach (Faussone-Pellegrini et al., 2005), cornea (Okada et al., 2011), spleen (Bertin et al., 2014) and kidney (Cortright et al., 2001).

Pain perception is a natural physiological warning mechanism that enables individuals to prevent harm and safeguard the tissue that has experienced trauma. Prolonged pain beyond the anticipated healing period may lead to an impaired state referred to as chronic pain, which affects a multitude of individuals globally. This condition can inflict long-term and incapacitating consequences, resulting in substantial disability and imposing significant health and socioeconomic burden.

Chronic pain is characterized by spontaneous pain such as burning or aching sensations, as well as pain that is induced by noxious (hyperalgesia) or non-noxious (allodynia) stimuli. The plasticity of neurons in pain pathways and circuits consisting of primary sensory neurons within the dorsal root ganglion (DRG) and trigeminal ganglia (leading to peripheral sensitization), as well as in pain-processing neurons located in the spinal cord and brain (resulting in central sensitization), may contribute to the development of chronic pain (Ji et al., 2003; Latremoliere and Woolf, 2009).

If well we are focused on TRPV1 channel in this contribution, it is important to note that peripheral sensitization, which is characterized by hypersensitivity and hyperexcitability of nociceptors due to tissue injury and inflammation, can be triggered by a diverse array of ion channels. These include thermoTRPs, sodium channels such a Nav1.7, Nav1.8, and Nav1.9 (Waxman et al., 1999; Amaya et al., 2000), and mechanosensitive piezo ion channels (Eijkelkamp et al., 2013).

### 1.2 TRPV1 channel sensitization

TRPV1 integrates of exogenous and endogenous signals, including pro-inflammatory mediators. A a polymodal receptor, TRPV1 senses multiple physical and chemical signals. Such sensations include burning, thermal pain, irritants, toxins derived from plants and animals (e.g., vanillotoxins, capsaicin, resiniferatoxin, and double-knot toxin). TRPV1 serves as a major receptor for painful stimuli from molecules created through metabolic activity or by receptors internally. This occurs during inflammation, nerve damage, chemotherapy, diabetes, or other pain-producing disorders, specifically via ligands such as lipoxygenase, endocannabinoids, and derivatives of arachidonic acid, linoleic acid, and lysophosphatidic acid. Many of the signals can lead to the sensitization of TRPV1 channels, which results in an increased response to low-intensity stimuli due to the channels’ greater plasma membrane availability and phosphorylation. Different signaling cascades such as bradykinin, prostanoids, nerve growth factor (NGF), and ATP can initiate this sensitization process. Furthermore, TRPV1 gating can be enhanced by protons, heat, and depolarization. Therefore, comprehending the regulatory mechanisms behind TRPV1 activation is vital for advancing new objectives for pain management and other medical treatments.

**FIGURE 3:**
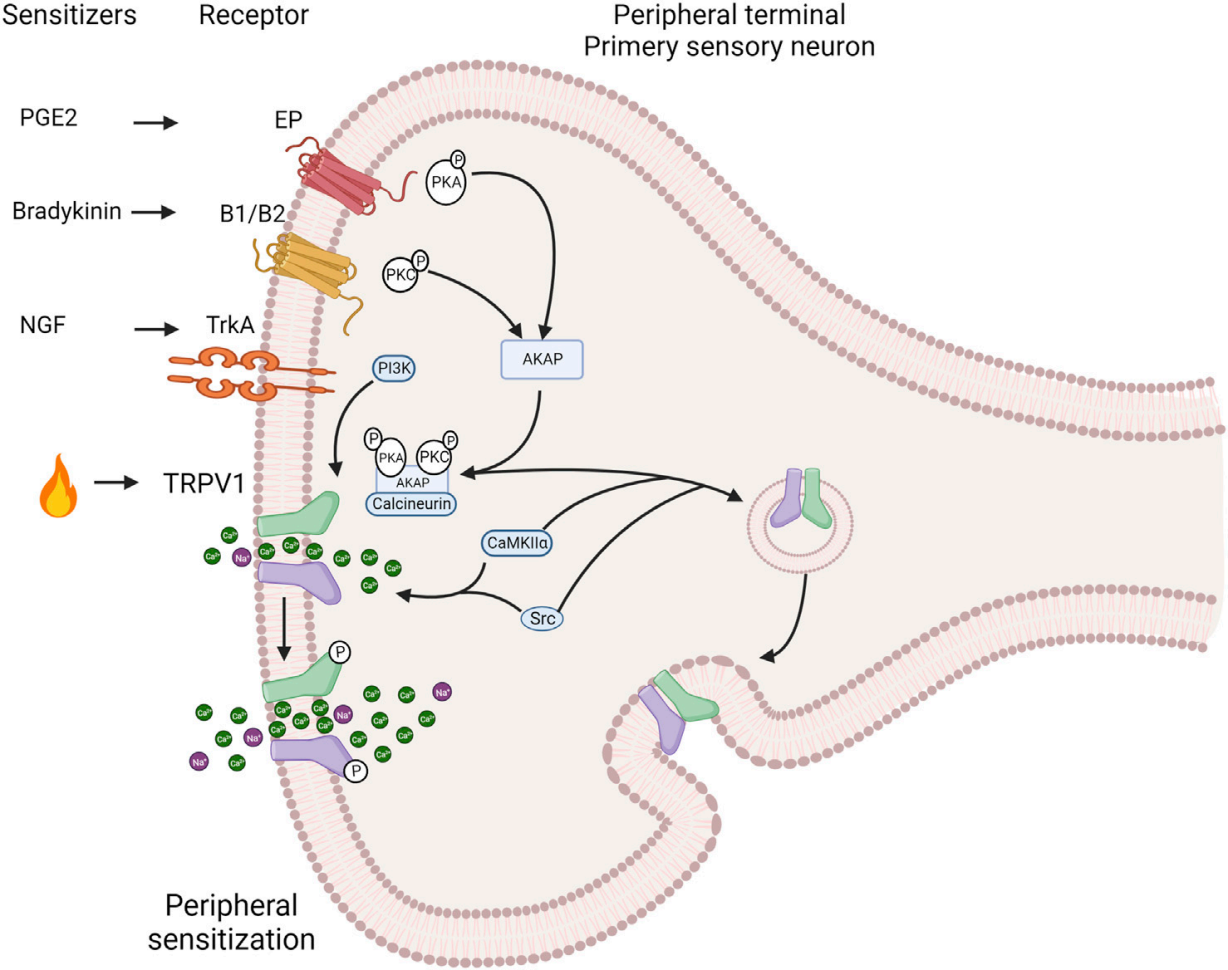
Induction of the TRPV1 channel deep sensitization by exogenous and endogenous signals. The activation of intracellular pathways related to G protein-coupled receptors (GPCRs) and protein kinases such as protein kinase C PKC), protein kinase A (PKA), calcium/calmdulin-dependent protein kinase II (CaMKII) and Src directly affects TRPV1 channel sensitization. During the inflammatory process nerve growth factor (NGF) is secreted, which binds to tropomyosin receptor kinase A (TrkA), leading to receptor dimerization and autophosphorylation. This, in turn, sensitizes TRPV1 through phosphorylation by phosphatidylinositol 3-kinase (PI3K) and the tyrosine kinase Src. Bradykinin activates B1 and B2 Gq-coupled receptors. This activation promotes PKCe phosphorylation and subsequent TRPV1 phosphorylation, resulting in channel sensitization. Similarly, PGE2 activates the Gs-coupled EP4 receptor, leading to sensitization through phosphorylation of TRPV1 by PKA. The scaffold protein AKAP 79/150 facilitates specific sensitization by phosphorylation by bringing macromolecular complexes, including PKA, PKC, and calcineurin, close to TRPV1. AKAP 79/150 bindsto the C-terminal region, specifically at residues D738, R740, C742 and V745. Inhibition of TRPV1 sensitization occurs when AKAP79/150-TRPV1 binding is disrupted. AKAP79/150 is involved in TRPV1 trafficking to the plasma membrane via PKC. The major site involved in trafficking control is S502. The tyrosine kinase Src can promote membrane trafficking by phosphorylating Y200, but does so independently of AKAP79/150 (Created with BioRender.com).

Injury or inflammation releases inflammatory mediators that lower the pain threshold. At least three pathways sensitize the TRPV1 channel, including cellular pathways related to G protein-coupled receptors (GPCRs) and several protein kinases such as PKC, PKA, CaMKII and Src that phosphorylate the channel (Figure 3). This produces a deep sensitization that triggers nociception, hyperalgesia and allodynia (Jung et al., 2004; Rosenbaum and Simon, 2007; Studer and McNaughton, 2010). The Gq-coupled B1 and B2 receptors activation by bradykinin decreases the threshold for temperature activation by promoting the PKCε (Cesare and Mcnaughton, 1996; Cesare et al., 1999; Numazaki et al., 2002; Bhave et al., 2003) phosphorylation and an increase in channel open probability under several activators. The metabolism of PIP_2_ by PLCβ could potentially impact the channel sensitization; however, this is a contentious issue because some reports suggest that PIP_2_ acts as a channel inhibitor (Chuang et al., 2001), while others indicate that it works as a channel activator (Stein et al., 2006; Lishko et al., 2007; Poblete et al., 2015). On the other hand, in sensory neurons, PGE2 activates the Gs coupled EP4 receptor leading to sensitization by triggering the phosphorylation of TRPV1 by PKA (Bhave et al., 2002; Distler et al., 2003). NGF sensitizes TRPV1 through PI3 kinase and the tyrosine kinase Src. This result in the phosphorylation of the channel and enhances its trafficking to the plasma membrane (Zhang et al., 2005; Stein et al., 2006).

The specificity and speed of channel sensitization via phosphorylation is facilitated by scaffolding proteins such as AKAP (A-kinase anchoring protein) that promote the encounter between kinases and the channel. AKAP 79/150 is indispensable in bringing macromolecular complexes, including PKA, PKC and calcineurin, near TRPV1 for their modulatory influence. In rodents (Zhang et al., 2008a; Brandao et al., 2012), AKAP79 and TRPV1 are co-expressed in small nociceptive sensory neurons. The sensitization of heat pain modulation, also referred as hyperalgesia, depends on the formation of a macromolecular signaling complex including the scaffold protein AKAP 79/150, TRPV1 and the enzymes PKC, PKA, and calcineurin enzymes (Zhang et al., 2008b; Jeske et al., 2009). AKAP79/150 binds to the C-terminal region of TRPV1, which facilitates channel phosphorylation and sensitization by bradykinin and PGE_2_. *In vitro* disruption of AKAP79/150 binding to TRPV1 or its binding mechanisms abolished TRPV1 sensitization, and a cell-permeant peptide that impedes the interaction of both proteins abolishes inflammatory hyperalgesia *in vivo* (Fischer et al., 2013). This anchoring protein is involved in trafficking TRPV1 to the plasma membrane through promotion of phosphorylation either by PKC and Src kinase (Morenilla-Palao et al., 2004; Zhang et al., 2005). Src has independent effects on TRPV1, compared to PKC or PKA (Numazaki et al., 2002; Bhave et al., 2003), and key residues in the C-terminal domain of TRPV1 identified for AKAP binding correspond to D738, R740, C742 and V745 (Fischer et al., 2013).

AKAP is capable to organizing various kinases and phosphatases into macromolecular complexes with their respective targets (Leiser et al., 1986; Bregman et al., 1989; Smith et al., 2006), such as PKA, PKC, and calcineurin (Coghlan et al., 1995; Klauck et al., 1996). Inhibiting pain sensitization through endogenous signaling presents a significant challenge due to complex macromolecular interactions of protein kinases and phosphatases, which regulate the phosphorylation status of the TRPV1 channel, as well as other ion channels like potassium and calcium channels and glutamate receptors. These protein complexes are also involved in various cellular functions and transduction pathways (Gao et al., 1997; Colledge et al., 2000; Altierab et al., 2002; Hoshi et al., 2003; Hoshi et al., 2005; Sandoz et al., 2006; Chai et al., 2007; Lu et al., 2007; Oliveria et al., 2007; Navedo et al., 2008; Fabbro et al., 2015), and the cessation of their activity could result in unintended consequences.

Heat hyperalgesia, a condition where injury or inflammation is lowers the threshold of heat-induced pain, is relieved in the absence of TRPV1 (Caterina et al., 2000; Davis et al., 2000). In rodent models the disruption of interaction between TRPV1 and AKAP 79/150 eradicates the inflammatory hyperalgesia (Fischer et al., 2013).

It has been reported that the activity of TRPV1 and Nav1.8 increases when phosphorylated by p38 MAP kinase. This results in peripheral sensitization when exposed to TNF and IL-1β in DRG neurons (Ji et al., 2002; Obata et al., 2004; Binshtok et al., 2008; Constantin et al., 2008; Matsuda et al., 2017). The elevated expression of TRPV1 prolongs the state of peripheral sensitization causing the transition from acute to chronic pain (Ji et al., 2002; Amaya et al., 2003, 2004). Additionally, the activation of p38 MAP kinase in C- and Aδ-fibers present in DRG neurons contributes to hypersensitivity to pain (Mizukoshi et al., 2013).

Phosphorylation has two distinct effects of on TRPV1 in whole-cell recordings: first it lowers the thermal threshold needed to activate TRPV1 and, second, it enhances the sensitivity of TRPV1 to other activating factors such as capsaicin, protons, and anandamide (Vellani et al., 2001). In addition, the TRPV1 channel is highly trafficked to the surface membrane following phosphorylation (Morenilla-Palao et al., 2004; Zhang et al., 2005), which plays a role in the development of heat hyperalgesia.

TRPV1 single-channel activity is significantly enhanced by PKC activation, in response to capsaicin. TRPV1 phosphorylation does not alter single-channel conductance and the increase in current that flowed through a single channel could be attributed to a higher probability to find the channel in the open state. The effect on the probability of the channel opening resembled that produced by an increase in capsaicin concentration (Studer and McNaughton, 2010), that is consistent with the observation that PKC activation produces an approximately 2-fold displacement in the capsaicin-induced TRPV1 dose-response curve in the whole-cell measurements (Vellani et al., 2001). PKC activation has two main effects on TRPV1. It shortens the time constant, decreases the occupancy of a closed state, and increases the occupancy of an open state. These changes ocurr with no significant difference in the time constant activation. PKC phosphorylates serine residues at positions 502 and 801 at C-terminus region. The first one is situated in a loop between transmembrane segments 2 and 3, which is close to the binding site for capsaicin, while the latter is in the C-terminal region. Phosphorylation boosts TRPV1 activity, by capsaicin and protons, whose binding sites are situated intracellularly and extracellularly, respectively (Jung et al., 1999; Jordt et al., 2000; Vellani et al., 2001; Jordt and Julius, 2002; Ryu et al., 2003). Phosphorylation of TRPV1 also enhances heat activation, but the location of the sensor for thermal stimulus is unknown.

Several neuropeptides are released from primary sensory nerve terminals into the peripheral terminals when C- or Aδ-fibers dorsal root ganglion (DRG) neurons are activated (Holzer and Maggi, 1998). These mediators directly or indirectly act on TRPV1, leading to nociceptors, immunocytes and endothelial cells activation and sensitization, strongly suggesting a significant of cross-talk between the nervous and immune systems. Indeed, these peptide mediators act on nociceptors, mast cells, immune cells, and vascular smooth muscle cells triggering inflammatory signaling cascades, edema and pain (Richardson and Vasko, 2002). This occurs because ATP and acidosis harm cells during injury. This, along with cytokines generated by immune cells, activate and sensitize sensory neurons, a condition known as neurogenic inflammation (Richardson and Vasko, 2002). Neurogenic inflammation is initiated by nerve stimulation, resulting in the release of neuropeptides and rapid plasma extravasation and edema, contributing to various pain conditions such as migraines, irritable bowel syndrome, vulvodynia, fibromyalgia, multiple sclerosis, painful bladder syndromes, arthrosis, airway inflammation conditions, and others (Richardson and Vasko, 2002; Birklein and Schmelz, 2008; Chiu et al., 2012). Neurogenic inflammation has been identified as prominently associated with inflammatory ailments such as asthma and psoriasis (Ji et al., 2018) in various clinical conditions. Even though eliminating nociceptors reduces neurogenic inflammation, it can have adverse effects because they can provide a favorable modulatory role to regulate inflammation in bacterial infections (Chiu et al., 2013; Chiu, 2018). Neurogenic inflammation is not solely provoked by the activation of peripheral C-fibers; it can also be initiated by local inflammation events or even through the activation of CNS primary afferents (Xanthos and Sandkühler, 2013; Ji et al., 2018). In fact, the CNS can experience neurogenic inflammation in response to neuroinflammatory events in the brain or spinal cord.

Neuroinflammation involves the activation of glial cells in dorsal root ganglia, spinal cord, and brain which leads to the production of proinflammatory cytokines and chemokines that drives peripheral and central sensitization (Ransohoff and Brown, 2012). On the other hand, neuroinflammation is a form of localized inflammation that can manifest in both the peripheral and CNS (Ji et al., 2014). It is characterized by heightened vascular permeability, infiltration of leukocytes, activation of glial cell, and exaggerated production of inflammatory mediators like cytokines and chemokines (Ji et al., 2014). The elevated permeability of the blood-brain barrier permits the infiltration of peripheral immune cells into the CNS. Neuroinflammation is linked chronic pain conditions, including postsurgical pain following major procedures such as amputation, thoracotomy, and mastectomy, as well as postoperative complications like delirium (Ji et al., 2018). Chronic pain persists beyond the resolution of clinical signs and inflammation symptoms, and neuroinflammation is closely associated with chronic pain states, potentially mediating and prolonging pain in human patients (Shi et al., 2012). Investigating the effects of various neuroinflammatory mediators on pain sensitivity in the pain neurocircuitry presents an intriguing research opportunity.

## 2 The molecular structure of TRPV1 channel

### 2.1 The TRPV1 channel: a close view into its architecture, agonist, and antagonist binding sites

**FIGURE 4:**
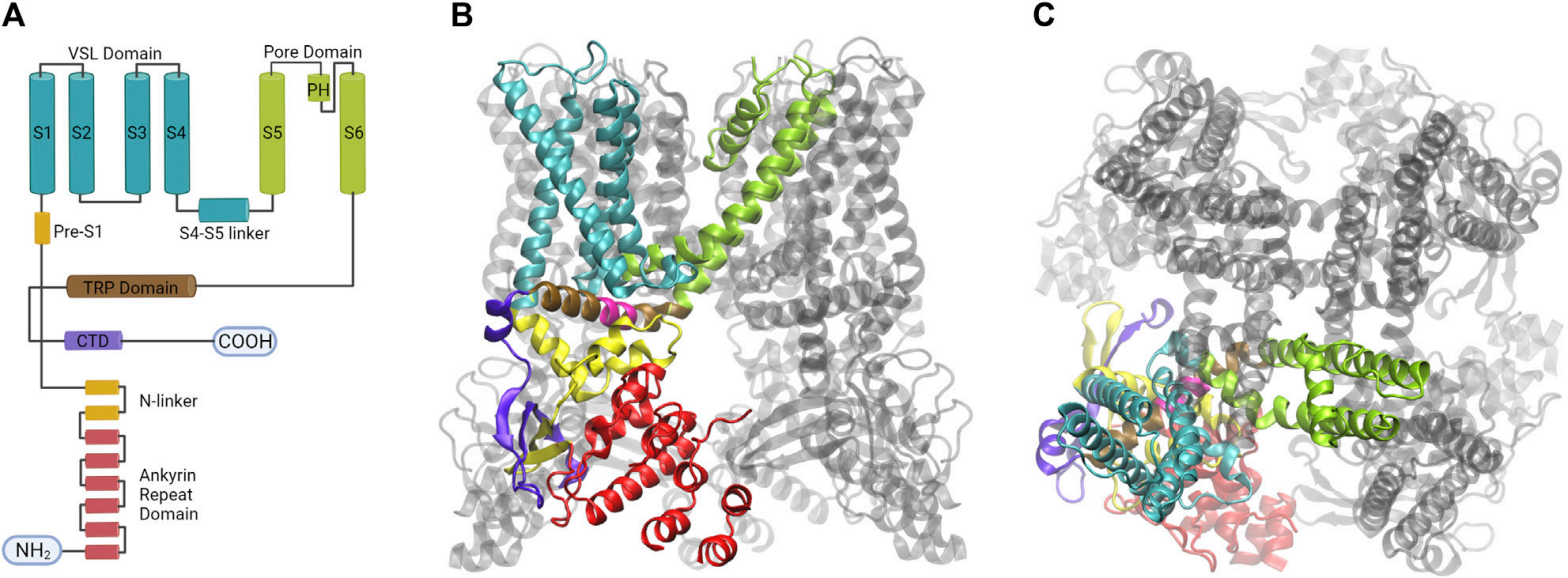
The homotetramer of the TRPV1 channel is organized by a quadruple-symmetric architecture. **(A)** Diagram illustrating the topology of the major structural domains found in a subunit of the TRPV1 channel. Each domain is color-coded: the voltage sensing like domain (cyan), the pore domain (lime), the TRP domain (brown), the ankyrin repeat domains (red), and the CTD domain (violet). These colors correspond to the colors of the ribbon diagrams shown to the right. **(B)** The image displays the tetrameric structure of the TRPV1 channel with one subunit highlighted from a lateral view **(C)** The architecture of the fourfold symmetry Is shown from a top view, revealing the interaction between S1- S4 and PD in a domain-swapped arrangement (Created with BioRender.com, Protein Data Bank: 7LPD).

The structure of rat TRPV1 obtained by single-particle cryogenic electron microscopy (cryo-EM) (Liao et al., 2013), confirmed the homotetrameric arrangement of the channel, in which each subunit consists of a protein containing 838 amino acids, composed of six transmembrane segments, intracellular ankyrin repeat domains ARD), and the amino- and carboxy-terminal domains, both facing the cytoplasm (Figure 4). Currently, the Protein Data Bank (PDB), contains 51 TRPV1 channel structures in different conformations at different temperatures and with different ligands, which have contributed to the understanding of channel gating since 2013 (Cao et al., 2013a; Liao et al., 2013; Gao et al., 2016; Kwon et al., 2021; Zhang et al., 2021; Kwon et al., 2022; Neuberger et al., 2023).

**FIGURE 5:**
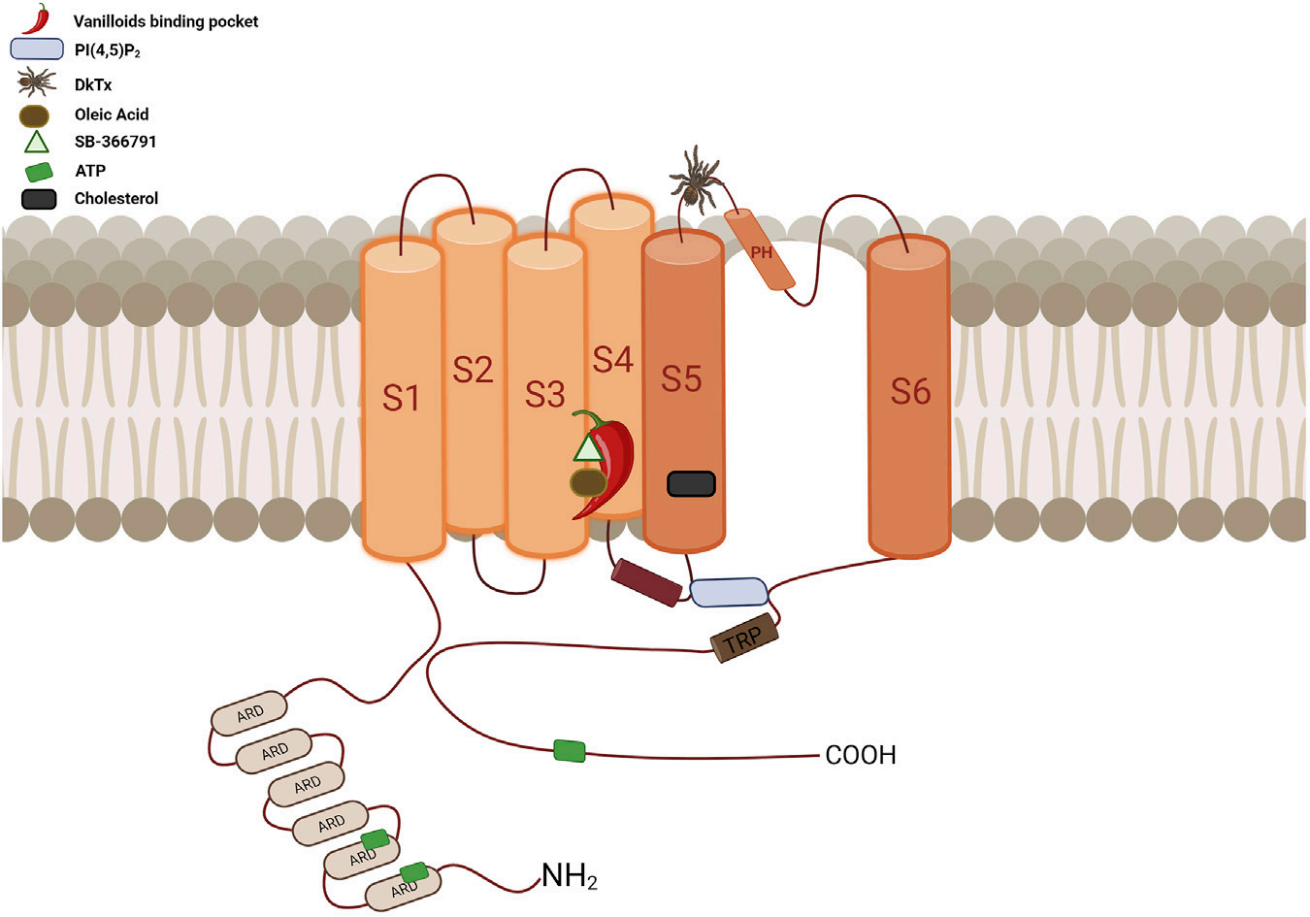
Diagrammatic overview of the TRPV1 channel’s topological arrangement and key binding sites location. The vanilloid binding site, represented by a chili pepper, is located between segments S3, S4, the S4-S5 linker, and the PD of the adjacent subunit. This site accommodates a variety of ligands such as CAP, RTX, CPZ, OA, PI, among others, all of which interact with at least one of these residues: Y512, S513, E571, T550, T551, and R557. The cholesterol interaction site is located between residues 579 to 586 at S5, specifically at the CRAC motif. ATP has three interaction sites: ANKR1, ANKR2 and the distal C-terminus. The binding site for PI(4,5)P_2_ is located between S3 and the S4-S5 linker, above the TRP domain. It interacts with residues R557 in the S4 segment, E570, R575, and R579 in the S4-S5 linker, and residue K694 of the TRP domain of an adjacent subunit. The DkTx site, represented by a spider, binds to the channel on the extracellular side, specifically in the outer pore region. It contacts residues at the top of the pore helix from one subunit and the outer pore loop of the adjacent subunit, interacting with residues I599, F649, A657, and F659 (Created with BioRender.com).

Segments S1 to S6 form the transmembrane domain (TMD), the TMD and ARD are connected by the coupling domain (CD), which includes a helix-loop-helix motif (HLHCD), a β-sheet (βCD), the pre-S1 helix (pre-S1CD), and a C-terminal domain (CTD) (Kwon et al., 2021). The N-terminal segment connects the sixth ankyrin repeat domain (ARD) to S1. The S1-S4 segments are considered to be the voltage sensor-like domain (VSLD), but the molecular nature of the voltage sensor has not been demonstrated, whereas the S5-S6 segments form the pore domain (PD). The pore helix acts as a pore radius regulator in the selectivity filter. In addition, the S1-S4 and PD interact with each other in a domain-swapped arrangement (Myers et al., 2008; Gao et al., 2016). The TRPV1 permeation pathway has two constrictions, one located at position G643 in the selectivity filter and the other located at residue I679 in the lower part of the S6 (Jara-Oseguera et al., 2019). The selectivity filter (SF) is located in the upper part of the pore between residues 643–646 and is composed of the residues glycine (G), methionine (M), glycine (G) and aspartate (D) (GMGD) whose backbone carbonyls or side chains point into the central pathway. The upper restriction does not act as a gate with defined open and closed states, but as a highly dynamic filter that allows permeation of small and large cations while contributing to the modulation of the lower gate (Jara-Oseguera et al., 2019; Zhang et al., 2021). In the C-terminal domain, the TRP box domain is oriented almost parallel to the membrane plane and is located below S6 and interacts with the S4–S5 linker, which facilitates coupling between different channel domains (Cao et al., 2013b; Liao et al., 2013) (Figure 5).

### 2.2 The vanilloid binding pocket: a heterogeneous binding site in the transmembrane domain

The vanilloid pocket, located in the TMD and facing the cytoplasmic leaflet of the membrane, consists of segments S3, S4 and the S4-S5 linker of one subunit and the pore domain (S5-S6) of the adjacent subunit. It owes its name to the favorable accommodation of compounds possessing the vanillyl group, such as capsaicin, a classical activator of the TRPV1 channel, and resiniferatoxin (RTX), the most potent activator described to date, both compounds of natural origin (Cao et al., 2013a). These vanilloid compounds appear to interact directly with the S4–S5 linker pulling it away from the central pore and consequently facilitating the opening of the lower gate (Gao et al., 2016). Indeed, the distance between the side chains is 5.3 Å in the unliganded state, whereas upon vanilloid binding, I679 rotates away from the central axis causing an expansion to 7.6 Å (Liao et al., 2013). Capsazepine, a synthetic vanilloid derived from capsaicin (Bevan and Geppetti, 1992), binds to this site and acts as a competitive inhibitor. In addition, several structures obtained by cryo-EM have shown that in the apo state the vanilloid pocket is occupied by a resident lipid, although the exact identity of the lipid has not been determined, which could be phosphatidylcholine, PI, PI(4,5)P_2_, and the displacement of these lipid molecules could lead to channel activation (Gao et al., 2016; Zhang et al., 2021; Neuberger et al., 2023). Although these ligands differ in structure and function, binding to the vanilloid pocket is favored by the caveolae spacing of the site and the rearrangement of the side chains of some key constituent residues, one of the most important being T511 (in the rat channel).

### 2.3 Capsaicin and RTX

In the vanilloid binding pocket, capsaicin adopts a “tail-up, head-down” configuration, where the tail interacts with the channel through Van der Waals forces (VDW) that contribute to binding affinity, while the interaction between the head and the channel is given by the hydrogen bonds between its vanillyl structure and residues Y512, S513, E571, T550, T551, and R557 within the TRPV1 channel (Yang et al., 2015a; Gao et al., 2016). After capsaicin binding, structural rearrangements occur to stabilize the S4-S5 linker in its outward (activated) conformation by “pulling” on E571 through hydrogen bonding and VDW contact with the linker. As the S4-S5 linker moves outward, S6 follows to open the activation gate. (Yang et al., 2015a). Φ-analysis has revealed that upon capsaicin binding, a conformational wave is initiated from the vanilloid binding pocket, propagates through the S4-S5 linker into the S6 bundle, and finally reaches the selectivity filter region, which opens the pore (Yang et al., 2018).

RTX is stabilized in the pocket by hydrophobic interactions between the diterpene ring of RTX and several residues including L515, V518 in S3 segment, M547 in S4, I573 in the S4-S5 linker and the L669 in S6 segment from the adjacent subunit. These interactions may be the reason for the high affinity binding of RTX. Also, RTX binding facilitates the interaction between R557 and E570, this interaction takes place in the space previously occupied by that of the PIP_2_ head group, thus moving the S4-S5 linker away from the central shaft, facilitating the opening of the lower gate (Gao et al., 2016; Zhang et al., 2021). As in the case of capsaicin binding, it has been proposed that upon RTX binding, TRPV1 undergoes a wave-like conformational propagation initiated in the vanilloid-binding pocket, first by opening of the S6 gate, then by opening of the SF, followed by reorganization of the PL and external pore (with further opening of the S6 gate and SF). This conformational wave is apparently due to additive and concerted conformational changes in many subdomains (Kwon et al., 2022). A recent study shows that RTX enhances structural fluctuations of TRPV1, but it has not been determined whether structural fluctuations are involved in channel gating (Sumino et al., 2023).

### 2.4 Lipids

Lipids play a crucial role in regulating TRPV1 activity. Specifically, phospholipids, fats, and steroids can modulateTRPV1 activity as agonists or antagonists (Rohacs, 2014; Zhai et al., 2020; Zhang et al., 2020; Manchanda et al., 2021; Rosenbaum and Islas, 2023).

It has been postulated that the activation of TRPV1 by annular lipids might facilitate allosteric communication between the vanilloid site and the peripheral cavities. The opening of TRPV1 necessitates the rotation of a conserved residue on the S6 segment from the S4-S5 linker towards the pore. This motion is linked to dehydration of the peripheral cavities between S6 and the S4-S5 linker. Hydration studies indicate that vanilloids and spider venom toxins bind to cause the ring lipids to adopt a distinctive “buried” conformation, which projects their hydrophobic tails into the peripheral cavities, encouraging dehydration and in consequence the opening of the channel (Gianti et al., 2019). This mechanism, which entails alterations in hydration, can explicate the lodging of molecules that serve as agonists or antagonists of the channel in the same pocket. These molecules may have varying associations with the pore, ultimately impacting the gating of the channel.

Phosphatidylinositol (PI), a resident lipid found within TRPV1, may have a significant influence on the allosteric activation of TRPV1 by vanilloid compounds and analogs. Research indicates that PI and capsaicin or resiniferatoxin all ocupy the same inter-subunit binding pocket in TRPV1, located between the voltage sensor-like domain and the pore domain. Capsaicin binding in the vanilloid pocket is potentially reliant on lipids through a sequential cooperativity mechanism.

In silico analysis suggests an initial, transient binding site with low affinity collaborates sequentially with a subsequent, dominant binding site with strong affinity. This cooperation may enable the release of the enclosed resident lipid (i.e., PI) for allosteric activation of TRPV1 by vanilloids or analogs through non-covalent interactions (Wang, 2021). High-resolution structures obtained through cryo-EM reveal a continued competition occurring within the vanilloid binding pocket (VBP) between vanilloid agonists and the resident regulatory PI lipid, utilizing a two-step process. Vanilloid agonists displace the regulatory PI lipid and compete for the aliphatic tail of PI. This is followed by the displacement of the inositol head group. It is worth noting that for the agonist to induce sufficient movement of S5, which is necessary for S6 movement and subsequently opening the lower gate of TRPV1, it must fully displace endogenous PI lipids in all subunits (Zhang et al., 2021). It has been hypothesized that heat destabilizes a hydrophobic cluster in the C-terminal domain, facilitating interactions between residues R739 and R743, and the corresponding adjacent linker domains. This leads to the breaking of hydrogen bonds between the linker domain and S2-S3 linker, which results in the formation of the vanilloid pocket. Then, the lipid in the VBP is displaced, and a new hydrogen bond is created between S4 and S5. This leads to S6 moving away from the ion channel, ultimately opening up the conduction pathway (Melnick and Kaviany, 2018).

The nature of TRPV1 interactions with membrane phosphoinositide molecules and whether these interactions facilitate or inhibit channel opening are controversial issues (Gunthorpe et al., 2004; Cao et al., 2013b; Gao et al., 2016; Zhang et al., 2021; Neuberger et al., 2023).

Several TRPV1 structures show a low density in the vanilloid binding pocket, identified as membrane phosphoinositide (PI) lipids. An allosteric mechanism has been proposed in which PI is released or displaced from the vanilloid binding pocket upon arrival of agonist at the site (Gunthorpe et al., 2004; Cao et al., 2013a; Gao et al., 2016; Zhang et al., 2021).

As mentioned above, it has been suggested that the TRPV1 channel may be negatively regulated by PI lipids as they inhibit TRPV1 by reducing sensitivity to chemical and thermal stimuli to prevent uncontrolled activation of TRPV1 in resting cells (Cao et al., 2013b). In the binding site where resident PI was identified, its branched acyl chains extend upward between S4 of one subunit and between S5 and S6 of an adjacent subunit, within a cleft that faces the interior of the membrane. The inositol ring is bounded on either side by S3 and the S4-S5 linker elbow, with the TRP domain below. The amino acid residue R557 (S4) can form polar interactions between the phosphate hydroxyl group at position 1 and E570 (S4-S5 linker) and a hydroxyl group at position 6 of the inositol ring; these interactions increase the stability of the PI at this site (Gao et al., 2016).

For PI(4,5)P_2_, a binding pocket has been proposed where the inositol ring is located between the S3 segment and the S4-S5 linker and above the TRP domain, where the presence of PI(4,5)P_2_ activates the channel (Poblete et al., 2015). The phosphate groups of PI(4,5)P_2_ interact with residues R557 in the S4 segment, E570, R575 and R579 in the S4-S5 linker, and residue K694 of the TRP domain of an adjacent subunit. Residue R701 may help stabilize the binding pocket once formed (Poblete et al., 2015). Several configurations of PI(4,5)P_2_ are in the vanilloid pocket and partially or fully displaced from it, suggesting that vanilloid binding displaces PIP_2_ from the pocket. Displacement of PI(4,5)P_2_ from the vanilloid pocket is essential for RTX-mediated activation, because if it is not fully displaced, the RTX-induced conformational change of S5 cannot be transmitted via the TRP helix to the lower gate in the S6 segment for TRPV1 channel opening (Zhang et al., 2021; Neuberger et al., 2023). It has been proposed that PI(4,5)P_2_ activates the channel through direct interaction with the proximal C-terminus (Ufret-Vincenty et al., 2011), and experiments with chimeras between TRPM8 and TRPV1 show that in both cases key residues in the TRP domain are involved in determining the apparent PI(4,5)P_2_ affinity of the channel (Brauchi et al., 2007).

### 2.5 Oleic acid (OA)

Oleic acid is a fatty acid found naturally in animals and vegetables and belongs to the omega-9 fatty acids. OA has been shown to be an allosteric inhibitor of the TRPV1 channel, and consistent with this, electrophysiological recordings showed that incubation of cleaved patches in OA 5 µM together with capsaicin 4 µM showed an 85% reduction in macroscopic current amplitude compared to those obtained in the presence of capsaicin 4 µM alone. In addition, 5 µM OA inhibited the current generated by noxious heat by 72% and by endogenous TRPV1 activators such as lysophosphatidic acid (LPA) by 95%, cPA by 79% and protons by 98%. Using competition assays, the researchers found that OA may compete with capsaicin for the same binding site, the vanilloid binding pocket. Molecular docking revealed that residue T550 forms hydrogen bonds with the carbonyl group of OA, as well as the residues Y511 and S512 is also important for OA binding. Mutagenic analysis using the Y511A-S512A-T550A triple mutant produces TRPV1 channels that are only 26% inhibited compared to 85% of wild-type rat TRPV1 channels (Morales-Lázaro et al., 2016).

### 2.6 Capsazepine

Capsazepine (CZP) is a synthetic compound derived from capsaicin that acts as a competitive inhibitor of the TRPV1 channel (Bevan et al., 1992) by exclusively occupying the vanilloid binding site. Binding of capsazepine to the channel promotes stabilization of the closed state removing the interaction between the side chains of residues R557 and E570, thus preventing channel opening. In addition, mutagenic analysis showed that the T551V mutation affects capsazepine-mediated inhibition (Yang et al., 2015a; Gao et al., 2016). Contrary to the effect of RTX, CZP decreases the structural fluctuation of TRPV1, but as mentioned above, it has not been established whether structural fluctuation is involved in channel opening (Sumino et al., 2023).

### 2.7 SB-366791

In 2004, the pharmaceutical company GlaxoSmithKline identified and characterized the compound SB-366791 (N-(3-methoxyphenyl)-4-chlorocinnamide) as a potent TRPV1 channel antagonist, as it inhibits the activation induced by various agonists such as capsaicin, protons, and noxious heat (Gunthorpe et al., 2004). Recently, the structure of the human TRPV1 channel in the presence of this compound was obtained, identifying four densities, one in each channel subunit, whose binding site corresponds to the vanilloid pocket. In the binding site, SB-366791 forms a hydrogen bond with residue Y511, an essential residue for capsaicin-mediated activation, and also forms hydrophobic interactions with residues L515 in the S3 segment and L547, T550, and L553 in the S4 segment. Mutagenic analysis showed that elimination of the hydrophobic interaction with residues L515, L547, and T550 renders the channel insensitive to SB-366791, and elimination of the hydrogen bond with residue Y511 decreases sensitivity to the compound, demonstrating that the vanilloid pocket corresponds to the binding site of the antagonist SB-366791 which acts as a competitive inhibitor (Neuberger et al., 2023).

### 2.8 Cholesterol

Cholesterol, a major component of plasma membranes, has been described to inhibit TRPV1 channel activity by binding to a cholesterol binding motif (CRAC) located between residues 579 to 586 in S5, specifically interacting with residue L585 in rTRPV1. The L585I substitution in rTRPV1 renders the channel sensitive to cholesterol (Picazo-Juárez et al., 2011).

### 2.9 Extracellular and intracellular binding sites

#### 2.9.1 DkTx

The Double Knot Toxin (DkTx), a toxin found in the venom of the Chinese Bird spider species, selectively and irreversibly activates TRPV1 channel. It is composed of 75 amino acids showing two inhibitory cysteine knot (ICK) motifs named knot1 (K1) and knot2 (K2) domains connected by a short linker (Bohlen et al., 2010; Bae et al., 2012). DkTx act as a bivalent ligand that engages the outer region of TRPV1, with each toxin moieties sitting at the subunit interfaces, contacting residues at the top of the pore helix from one subunit and the outer pore loop proximal to the S6 segment from the neighboring subunit, interacting with residues I599, F649, A657, F659 (Cao et al., 2013b). Mutations of these residues to alanine turned out TRPV1 to be DkTx insensitive (Bohlen et al., 2010). A comparative analysis of the DkTx bound and unbound structures indicates that binding of DkTx promotes changes in the VSLD of each subunit, affecting their relative arrangements. Furthermore, when DkTx is fully coupled to the channel in the open state, the VSLD is tilted and twisted, acting as a major module that allosterically couples changes in the outer pore to the lower gate. As a result, all these changes open the constrictions observed in the apo structure, both in the SF region and in the intracellular gate (Bae et al., 2016; Zhang et al., 2021).

#### 2.9.2 ATP

ATP acts as a positive allosteric modulator that directly prevents tachyphylaxis to repeated applications of capsaicin. The binding site for ATP was found in the ARD specifically located in ankyrin repeats 1-3, where the triphosphate interacts with residues R115 in inner helix 1 and K155 and K160 in inner helix 2, while residues L163 also in inner helix 2 and Y199 in finger 2 were found to be intercalated with the adenine base. The N6 amine of adenine also interacts with Q202 in finger 2 and E210 in inner helix 3. Mutagenic analysis of residues K155A, K160A, or Y199A/Q202A showed little tachyphylaxis even in the absence of ATP (Lishko et al., 2007). In addition, mutation of K735, in the distal C- terminal, resulted in a strong inhibition of ATP binding (Grycova et al., 2007).

## 3 Temperature sensing

The physical fundaments of thermosensitivity in thermoTRP channels is unknown, although several theoretical frameworks have been proposed to explain this remarkable feature of these transmembrane proteins. The characterization of the activity of thermoTRP channels at different temperature ranges has been accurately determined by electrophysiology under temperature-controlled conditions.

Due to the polymodal nature of these ion channels and species-specific differences in thermal sensitivities, the search for the temperature sensor in thermoTRP channels has been challenging. Reconstitued ThermoTRP channels in artificial membrane systems, confirmed their intrinsic temperature sensitivities (Tominaga et al., 1998; Saito and Shingai, 2006; Zakharian et al., 2010; Yao et al., 2011; Sardar et al., 2012; Saito and Tominaga, 2015; Saito et al., 2016; Saito and Tominaga, 2017; Hori and Saito, 2023). The existence of a temperature sensor has implicit the presence of a domain or residues containing the structural elements for temperature detection, in analogue manner as the voltage sensor in voltage-gated K^+^ channels. Evidence of the existence of structurally defined modules in thermoTRP channels harboring temperature sensors comes from experiments that exchanged entire modules between channels with different thermal sensitivities (Brauchi et al., 2006; Cordero-Morales et al., 2011; Gracheva et al., 2011; Yao et al., 2011; Zhong et al., 2012).

Four members of the TRPV subclass are activated by temperature. TRPV1 is activated by temperatures above 42°C (Caterina et al., 1997, 1999; Smith et al., 2002; Watanabe et al., 2002; Xu et al., 2002; Patapoutian et al., 2003a; Moqrich et al., 2005; Voets et al., 2005; Togashi et al., 2006; Song et al., 2017; Vandewauw et al., 2018; Paricio-Montesinos et al., 2020). In fact, several publications demonstrate that TRPA1 is a noxious cold receptor (Bandell et al., 2004; Sawada et al., 2007; Karashima et al., 2009; Kremeyer et al., 2010), but others indicate that this channel is not activated by cold (Jordt et al., 2004; Bautista et al., 2006; Zurborg et al., 2007; Cordero-Morales et al., 2011) temperature (Moparthi et al., 2016).

Structural modules involved in temperature sensing are thought to be located in the N- and C-termini (Brauchi et al., 2006; Yao et al., 2011), pore turret, and pore (Grandl et al., 2010; Yang et al., 2010) of the TRPV1 channel. However, it was shown that deleting the entire pore turret of the channel (residues 604–626), it remains activated by heat (Liao et al., 2013). The thermal sensitivities of TRPV1 (heat-activated) and TRPM8 (cold-activated), are exchanged by swapping the C-terminal domains between them (Brauchi et al., 2006). In addition, a region between amino acids Q727 and W752 of TRPV1 could convert TRPM8 into a warm-activated channel (Brauchi et al., 2007). In both TRPV1 and TRPV2 channels, is thought to be that the membrane proximal domain (MPD) is a key component of the temperature sensing mechanism (Yao et al., 2011). Ground squirrels and camels exhibit high tolerance to environmental heat, enabled by the expression of TRPV1 channels with a deeply reduced temperature sensitivity (Laursen et al., 2016). This is caused by a single mutation of N190 in ground squirrels and camels, which is a serine residue in rat, in the first ankyrin repeat domain (ARD). In fact, heat sensitivity is completely restored by replacing this amino acid in the insensitive clones (Laursen et al., 2016).

Temperature dependence in chemical systems, including ion channels, can be quantified by Q_10_, a dimensionless factor that represents the change in reaction rate for a 10° change in temperature (Ito et al., 2015). In thermodynamics, a high value of Q_10_ indicates the presence of a large activation energy (i.e., large enthalpy) over a short period of time during the transition process between the closed and open states of the channels (Ito et al., 2015). In ion channels Q_10_ and thermodynamic state functions can be estimated from voltage-activated currents obtained at different temperatures as described before (Baez et al., 2014; Feng, 2014; Castillo et al., 2018). An increase in temperature speeds up the activation of most proteins. In ion channels that are not temperature-sensitive, the gating processes exhibit a Q_10_ of ∼3 (Hille, 1987). It was suggested that a thermoTRP channel must possess a Q_10_ ≥ 5 to be classified as a temperature-sensitive TRP channel (Voets, 2012). Accordingly, temperature changes trigger significantly high Q_10_ values in cold and heat receptors, which have Q_10_s ≥ 25 (Liu et al., 2003; Brauchi et al., 2004; Yao et al., 2010; Raddatz et al., 2014).

The temperature the channel deactivation kinetics of temperature-gated currents displays the temperature dependence in cold receptors, whilethe temperature-dependent process in heat receptors is reflected in the channel activation kinetics (Brauchi et al., 2004; Voets et al., 2004; Yao et al., 2010; Baez et al., 2014; Raddatz et al., 2014). In the case of the TRPV1 channel, the enthalpy change related to channel activation is ∼100 kcal/mol, with a corresponding Q_10_ of ∼50 for its activation (Yao et al., 2010). Compared to that, deactivation of TRPM8 channels results in a total enthalpy change of ∼46 kcal/mol, which corresponds to a Q_10_ of ∼33 (Raddatz et al., 2014). These large enthalpy changes are counteracted by significant entropy changes, enabling the closed-open equilibrium to be reversible.

Electrophysiological recordings have revealed details of the mechanisms of activation of TRPV1 by capsaicin, heat and low pH (Premkumar et al., 2002; Hui et al., 2003; Liu et al., 2003; Ryu et al., 2003). Single-channel measurements and analysis have yielded significant insights into the behavior and energetics of TRPV1 activity as temperature increases, facilitating a more comprehensive of channel functions. Since thermoTRP channel activity involves substantial entropic and enthalpic changes, the assessment of opening energy has proven essential. However, it is experimentally challenging to measure this energy when a high temperature (≥40°C) is necessary for full channel activation (Nagy and Rang, 1999; Vyklický et al., 1999; Welch et al., 2000; Liu et al., 2003; Brauchi et al., 2004; Voets et al., 2004). Single-channel analysis has revealed that the gating process based gating on temperature involves complex kinetics with several closed and open states (Liu et al., 2003). The effect of temperature is mainly noticeable in longer closures, while short closures and openings exhibit little dependence on temperature. The observed kinetic characteristics and voltage gating modeled at varying temperatures align with the notion that channel opening is triggered by temperature and that the single-channel long closures are the microscopic origin of the activation time course (Voets et al., 2004). The duration of bursts in single-channel activity is significantly temperature-dependent. Nevetherless, the open and closed components of the bursts are impervious to temperature. This implies that the apparent temperature sensitivity of the bursts is a secondary effect. It emerges from a shift in the occupancy of populations rather than fluctuations in opening and closing rates (Liu et al., 2003).

To date, at least three different mechanisms have been proposed to explain the exquisite temperature sensitivity of thermoTRP channels: i) if ΔH and ΔS are temperature independent, the balance between large changes in enthalpy and entropy drives the cold or heat sensitivity (Liu et al., 2003; Brauchi et al., 2004; Voets et al., 2004); ii) another potential mechanism is based on changes in heat capacity between the closed and open states (Clapham and Miller, 2011), which requires that a thermoTRP channel can be activated by heat and cold and exhibits an inverted bell-shaped curve (dual thermosensitivity) in the Po-temperature curve (Feng, 2014), a situation that has only been tested experimentally in hTRPA1 (Moparthi et al., 2016); and iii) allosteric thermoTRP channel models with intrinsically temperature-dependent coupling constants can also generate heat- and cold-activated channels (Jara-Oseguera and Islas, 2013).

Despite the numerous cryo-EM structures acquired for TRP channels, information regarding how temperature induces rearrangements in thermoTRP channels is lacking. As a result, the molecular foundation for the gating by temperature remains elusive.

## 4 Pharmacological compounds targeting the TRPV1 channel

The role of TRPV1 channels as pain receptors in physiological and pathophysiological processes represents a promising alternative for the therapeutic development of new compounds with analgesic functions. Although narcotics (opioids) have been used effectively as analgesics for several decades, their use is associated with addiction and brain damage, which creates several work and social problems (Hser et al., 2015). The identification of the TRPV1 channel as a pain receptor has led to intense biomedical research over the last 20 years in the search for new TRPV1 receptor agonists and antagonists to be used as analgesics without the undesirable side effects of opioids. Currently, a variety of exogenous ligands have been developed and characterized for targeting TRPV1 (Table 1). This include various natural compounds, synthesized molecules, and toxins derived animal, fungal, plant, and bacterial venoms. In particular, a classic agonist of TRPV1 is capsaicin, whose therapeutic effect has led to the development of drugs for pain relief. The prolonged exposure of TRPV1 channel to capsaicin activates TRPV1 channel and then produce channel desensitization, by Ca^2+−^dependent mechanisms, which reduce the channel activity, producing the analgesic effect. This has led to the development of the capsaicin transdermal patch (8%), indicated for moderate musculoskeletal and peripheral neuropathic pain, which is applied directly to the side of the pain. However, when TRPV1 channels are sensitized in inflammatory pain conditions the use of agonists is limited due to the side effects caused by activation of the channel, such as increased purgency and neurotoxic effects. In this context, the use of antagonists in the development of pain medications has been considered to have greater therapeutic advantages than TRPV1 agonists (Caterina, 2008; Anand and Bley, 2011).

**TABLE 1:** TRPV1 channel agonists and antagonists.

Name	Ligand	Group	Origin	Function	IC_50_/EC_50_ values	Binding site	References
Capsaicin	agonist	vanilloids	*Capsicum* spp. (Hot pepper)	Increases intracellular Ca^2+^ concentrations in HEK293 cells. Analgesic effect.	EC_50_ = 0.1 μM	vanilloid binding pocket	Caterina et al. (1997)
Resiniferatoxin (RTX)	agonist	vanilloids	*Euphorbia resinifera*	increases intracellular Ca^2+^, the most potent agonist of TRPV1	EC_50_ = 1–2 nM	vanilloid binding pocket	Szallasi and Blumberg (1989), Winter et al. (1990)
			*Einhorbia poissonii*				
6-Gingerol	agonist	vanilloids	*Zingiber officinale* (ginger)	Similar to capsaicin, increases intracellular Ca^2+^ in cultured DRG neurons. Decreases the cancer-promoting effect.	EC_50_ = 2.9 μM	vanilloid binding pocket	Dedov et al. (2002), Geng et al. (2016), Yin et al. (2019)
8-Gingerol	agonist	vanilloids	*Z. officinale*	Similar to capsaicin, increases intracellular Ca^2+^ in cultured DRG neurons.	EC_50_ = 2.09 μM	vanilloid binding pocket	Dedov et al. (2002), Yin et al. (2019)
10-Gingerol	agonist	vanilloids	*Z. officinale*	increases intracellular Ca^2+^ and modulates adrenaline secretions.	EC_50_ = 2.0 μM	vanilloid binding pocket	Iwasaki et al. (2013), Yin et al. (2019)
Dihydrocapsiconiate Capsiconiate	Agonist	vanilloids	*Capsicum baccatum*	Increases Ca^2+^ influx in HEK293 cells expressing rTRPV1.	EC_50_ = 4.2 μM		Kobata et al. (2008)
					EC_50_ = 3.2 μM		
6-Paradol	agonist	vanilloids	*Aframomum melegueta*	Increases Ca^2+^ influx in HEK293 cells expressing rTRPV1.	EC_50_ = 0.7 μM	vanilloid binding pocket	Riera et al. (2009), Fajrin et al. (2020)
R-(−)-carvone	agonist	terpenoids	*Mentha* spp.	Increases Ca^2+^ influx in HEK293 cells expressing rTRPV1.	EC_50_ = 1.3 mM		Gonçalves *et al.* (2013)
Miogatrial	agonist	terpenoids	*Zingiber mioga*	Dose-dependent Ca^2+^ influx increases in HEK293 cells expressing rTRPV1.	V EC_50_ = 0.63 mM		Iwasaki et al. (2009)
isopropyl ITC, sec-butyl ITC, allyl ITC, benzyl ITC, phenylethyl ITC, p-hydroxybenzyl ITC, 3-methylthiopropyl ITC	agonist	sulfur containing compounds	Wasabi (*Wasabia japonica*) & horseradish (*Cochlearia armoracia*)	Increases Ca^2+^ influx in HEK cells expressing rTRPV1.	isopropyl ITC: E50 = 100 μM		Terada et al. (2015)
					sec-butyl ITC: E50 = 100 μM		
					allyl ITC: E50 = 160 μM		
					benzyl ITC: E50 = 223 μM		
					phenylethyl ITC: E50 = 180 μM		
					p-hydroxybenzyl ITC: E50 = 170 μM		
					3-methylthiopropyl ITC: E50 = ?		
docosahexaenoic acid	agonist	fatty acids	animal sources (eggs, meat)	Increases Ca^2+^ influx in HEK293 cells expressing rTRPV1.	EC_50_ = 36.0 μM		Matta et al. (2007)
Cannabichromene, Cannabidiol, Cannabidiol acid, Cannabigerol, Cannabigivarin	agonist	cannabinoids	*Cannabis sativa*	Increases Ca^2+^ influx in HEK293 cells expressing hTRPV1.	Cannabichromene: EC_50_ = 24.2 μM	hydrophobic pocket located between S5 and S6 helices	De Petrocellis et al*.* (2011)
					Cannabidiol: EC_50_ = 1 μM		
					Cannabidiol acid: EC_50_ = 19.7 μM		
					Cannabigerol: EC_50_ = 1.3 μM		
					Cannabigivarin: EC_50_ = 2 μM		
RhTx	agonist	Peptide toxin	*Scolopendra subspinipes mutilans*	Increases Ca^2+^ influx in HEK293 cells expressing rTRPV1.	EC_50_ = 500 nM		Yang et al. (2015a)
DkTx	agonist	Peptide toxin	*Ornithoctonus Huwena* (Earth Tiger Tarantula)	stabilizes the open state of the channel, as it is an irreversible activator.	EC_50_ = 0.23 μM	DkTx site	Bohlen et al. (2010), Zhang et al. (2021)
BmP01	agonist	Peptide toxin	*Mesobuthus martensii* (Chinese scorpion)	Intracellular Ca^2+^ influx increases, and the effective concentration is pH dependent.	EC_50_ = 131.8 μM		Yang et al. (2017)
Curcumin	antagonist	vanilloids	*Curcuma longa*	blocks capsaicin-induced thermal hyperalgesia by inhibiting TRPV1 currents expressed in trigeminal ganglion neurons such as HEK 293 cells.	IC_50_ = 67 nM		Yeon et al. (2009)
Cochinchinemin A	antagonist	flavonoids	*Dracaena cochinchinensis* (dragon’s blood resin)	inhibits capsaicin induced TRPV1 currents in DRG neurons	IC_50_ = 46.64 μM		Wei et al. (2013)
Cochinchinemin B	antagonist	flavonoids	*D. cochinchinensis* (dragon’s blood resin)	inhibits capsaicin induced TRPV1 currents in DRG neurons	IC_50_ = 718.32 μM		Wei et al. (2013)
Eriodictyol	antagonist	flavonoids	*Eriodictyon californicum*	Shifts RTX binding and inhibits capsaicin mediated Ca^2+^ influx. Prevents the development of oxidative stress in the lumbar spinal cord.	IC_50_ = 47 nM		Rossato et al. (2011)
Baicalin	antagonist	flavonoids	*Scutellaria baicalensis*	Decreases TRPV1 mRNA expression in DRG neurons. Decreased intracellular calcium fluorecent activity evoked by capsaicin.			Sui et al. (2010)
Evodiamine	partial agonist and antagonist	alkaloids	*Evodia rutaecarpa*	Activates currents in HEK293 cells. Inhibits capsaicin- and proton-induced currents	EC_50_ = 0.6 μM		Wang et al. (2016)
Pellitorine	antagonist	alkaloids	*Tetradium daniellii*	Inhibits intracellular Ca^2+^ influx evoked by capsaicin	IC_50_ = 154 μg/mL		Oláh et al. (2017)
Eucalyptol	antagonist	terpenoids	*Eucalyptus globulus* (Tasmanian bluegum)	Reduces capsaicin-induced currents in orofacial pain.			Melo Júnior *et al.* (2017)
Albaconol	antagonist	triprenyl phenols	*Albatrellus* spp. (fungus)	Weakly inhibits TRPV1 currents in DRG neurons.	IC_50_ = 17.0 μM		Hellwig *et al.* (2003)
Grifolin					IC_50_ = 30.0 μM		
Neogrifolin					IC_50_ = 7.1 μM		
Linoleic acid	antagonist	fatty acids	vegetable oil, avocado, canola, canola, carthamus.	Inhibits TRPV1 activity, such as the response to pain and itching.			Morales-Lázaro et al. (2016)
linolenic acid	antagonist	fatty acids	animal sources (eggs, meat)	Inhibits intracellular Ca^2+^ influx evoked by capsaicin			Matta et al. (2007)
eicosapentaenoic acid	antagonist	fatty acids	Fish	Inhibits TRPV1 activity, such as the response to pain	EC_50_ = 29.7 μM		Matta et al. (2007)
PnTx3-5	antagonist	Peptide toxin	Phoneutria nigriventer (Armed spider)	Inhibits intracellular Ca^2+^ influx evoked by capsaicin			Rita Pereira et al*.* (2020)

The effects of capsaicin on the activation of TRPV1 can be potentiated by acidic pH (Caterina et al., 1997), consistent with the synergism of protons and the heat receptor in the excitation of pain pathways (Bevan and Geppetti, 1994). Subcutaneous, oral, and central administration of TRPV1 agonists induces hypothermia (Jancsó-Gábor et al., 1970; Hori, 1984; Szelényi et al., 2004; Gavva et al., 2007a; Yoshida et al., 2016; Inagaki et al., 2019), suggesting a role in thermal homeostasis. Administration of agonists of capsaicin rinvanil induces hypothermia (Jancsó-Gábor et al., 1970; Muzzi et al., 2012), whereas administration antagonist blocks agonists-induced hypothermia and also causes an increase in temperature (hyperthermia) (Gavva et al., 2007b; Gavva, 2008). Consistently, the TRPV1-deficient mouse confirmed its involvement in painful thermal sensation and hyperalgesia (Caterina et al., 1997). In addition, TRPV1 KO mice undergo hyperthermia and heat loss when exposed to warm, suggesting that central expression of TRPV1 is relevant in thermosensation and thermoregulation (Yonghak et al., 2020).

Therefore, the search for specific TRPV1 channel antagonists has been a major focus of research in recent years. However, the use of blockers or inhibitors of TRPV1 present some concerns that emerged from clinical trials such as hyperthermia caused by the peripheral block of the channel and an increase in the threshold for temperature sensitivity, which can cause burnings (Gavva et al., 2007a; Papakosta et al., 2011; Vay et al., 2012). These has slow down the develop of TRPV1 blockers as analgesics.

## 5 Physiology and pathophysiology related to TRPV1

As described above, TRPV1 is predominantly distributed in the primary afferent neurons of the somatosensory system and are known to be the primary input molecular substrate leading to pain perception (Caterina et al., 1997). Although the first processes identified for TRPV1 were nociception and thermosensation, research has been conducted to elucidate the molecular mechanisms underlying other physiological and pathophysiological processes. Involved in nociceptive, inflammatory, and neuropathic pain, TRPV1s are activated by noxious stimuli (heat or chemical stimulation) and allow Ca^2+^ influx, allowing local depolarization by opening voltage-dependent sodium channels and generating afferent action potentials that allow information to propagate to central nervous system (Patapoutian et al., 2009b). In this context, a human genetic variant of TRPV1 has recently been identified that implicates residue K710 as a critical site in the control of TRPV1 nociception (He et al., 2023).

Activation of TRPV1 in the organ of Corti and spiral ganglion cells suggests hearing loss due to increased generation of reactive oxygen species (ROS) in the cochlea (Zheng et al., 2003; Mukherjea et al., 2008). Similarly, salicylate has been described to induce tinnitus via TRPV1 (Kizawa et al., 2010). The distribution of TRPV1 in the brain affects neuronal activity by playing a fundamental role in synaptic transmission, neurotransmitter release, and plasticity (Li et al., 2008; Maione et al., 2009). In fact, increased expression of TRPV1 channels has been found in the hippocampus in epileptic conditions, suggesting an important role in this pathophysiology (Bhaskaran and Smith, 2010; Saffarzadeh et al., 2016). TRPV1, located in the cardiovascular system, has been shown to modulate vasoconstriction, regulate blood flow, and increase systemic blood pressure (Phan et al., 2020). TRPV1 activity reduces lipid storage, which is a promising therapeutic tool for atherosclerosis, as the use of copper sulfide (CuS) nanoparticles targeting TRPV1 channels in vascular smooth muscle cells helps to attenuate fat accumulation (Gao et al., 2018). TRPV1, expressed in islet β cells and nerve fibers innervating the pancreas, promotes insulin release by increasing Ca^2+^ concentration, which regulates appetite and body weight (Razavi et al., 2006; Waluk et al., 2013; Jeong et al., 2018), and modulates type 1 and type 2 diabetes by participating in adiponectin and leptin signaling (Derbenev and Zsombok, 2016; Liu et al., 2023). In the bladder, TRPV1 channel expression is distributed throughout the lower urinary tract structures and plays an important role in the micturition reflex (Birder et al., 2002; Daly et al., 2007; Mistretta et al., 2014). Similarly, TRPV1 expression in the respiratory tract is involved in the cough reflex (Adcock, 2009; Lee et al., 2011). In the male reproductive system, TRPV1 has been implicated in sperm calcium homeostasis (Xiao and Chen, 2022).

TRPV1 channels have recently been found to play an important role in cancer development and tumorigenesis, in various types of cancer. Recently, it has been reported that expression of TRPV1 in gastric cancer suppresses tumor development (Gao et al., 2020). In addition, capsaicin activation in breast cancer inhibits cell growth and induces apoptosis, thereby improving prognosis (Weber et al., 2016). Similar cases have been reported in thyroid cancer (Xu et al., 2018), prostate cancer (Czifra et al., 2009), pancreatic cancer (Huang et al., 2020), skin cancer (Bode et al., 2009), and bladder cancer (Mistretta et al., 2014). The regulation of TRPV1 in metastasis has recently been suggested to play a possible inhibitory role in migration and proliferation through Ca^2+^- activated pathways triggered by ion flux across the channel (Li et al., 2021).

## 6 *In silico* tools for TRP channel drug discovery/repositioning

The foregoing strongly supports the search for compounds capable of regulating TRPV1 channels to treat various pathophysiological and pathological conditions. Although clinical trials are the most expensive phase of drug development, significant opportunities for time and cost savings lie in the earlier stages of discovery, also known as Computer-Aided Drug Discovery (CADD). These early stages include identifying potential drug targets, designing, and synthesizing compound libraries, conducting *in vitro* and *in vivo* experiments, and optimizing drug candidates for further evaluation. By prioritizing efficient strategies in these early stages, pharmaceutical companies can streamline the drug development process, reduce costs, and accelerate the availability of safe and effective therapies (Sadybekov and Katritch, 2023).

### 6.1 Leverage the wealth of 3D protein structures for targeted CADD

The exponential growth of protein data enabled by cryo-electron microscopy (cryo-EM) technology has provided remarkable insights into the 3D structures of ion channels, including the transient receptor potential (TRP) family (Fernandez-Leiro and Scheres, 2016; Nannenga and Gonen, 2019). While Cryo-EM has successfully resolved the majority of *Homo sapiens* TRP channels, offering detailed insights into their structural peculiarities, such as modulation by various biologically relevant ligands and the effects of different physicochemical conditions on their activity, it’ essential to note that cryo-EM data provides 3D snapshots of a channel’s conformations. In most cases, these snapshots represent a single physiologically important conformation. Recent advances in artificial intelligence, exemplified by the groundbreacking AI system AlphaFold2 (AF2) during CASP14, have transformed the field of protein structure prediction (Pahari et al., 2019; Jumper et al., 2021; Pereira et al., 2021; Marcu et al., 2022; Varadi and Velankar, 2022). The ability of AF2 to predict protein structures has profound implications for target identification and validation tasks in the CADD process, even for targets such as the TRP superfamily with limited structural information for some members or physiologically relevant conformations.

The functional relevance of membrane proteins, such as the TRP superfamily, in all domains of life lies in their ability to adopt multiple interconverting conformational states by crossing kinetic barriers (Liu and Montell, 2015; López-Romero et al., 2019). In general, these multiple states are intricately linked to the specific function of the protein and are crucial for understanding the gating mechanism (Boehr et al., 2009; Cournia et al., 2015; Stein and McHaourab, 2022). Recognizing the importance of these conformational states is particularly important in CADD. By targeting specific conformations, researchers can develop drugs that selectively modulate the activity of the protein to promote desired therapeutic outcomes. In this context, Saldaño T. et al. report that AF2 predominantly predicts the holo form in about 70% of cases, showing limitations in capturing the observed conformational diversity equally for holo and apo conformers, suggesting the potential use of predicted local model quality scores to infer ligand-binding induced conformational changes.

Nevertheless, the integration of stochastic subsampling into multiple sequence alignment within AF2 has shown potential in aiding the creation of models that aim to approximate the multiple conformations of membrane proteins (Del Alamo et al., 2022). These conformations are posited to bear resemblance to native structures, acknowledging that our current methodologies, including cryo-EM and crystallography, may not provide a definitive picture of native states, as alluded to by recent discussions in the field (Shoemaker and Ando, 2018). In addition, it has been tested that the ability of AF2 to predict multiple conformations for a given protein sequence holds potential for engineering protein and mutant induced activity (Cummins et al., 2022). Another success derived from AF2 is the recent update of the Membranome Database 3.0, which incorporates models generated by AF2 and validated with experimental information (Lomize et al., 2022).

### 6.2 Exploring binding sites in TRP channels with CADD

Identification of primary ligand-binding sites is crucial in CADD for targeting proteins. These sites are where ligands directly interact with enzymes, competing with substrates or cofactors to alter or change protein activity. For TRP channels, these pockets/sites are where direct modulation of channel gating occurs exemplified by the vanilloid site in TRPV1 that bind capsaicin (Yang et al., 2015b; Darré and Domene, 2015). Understanding the structural and functional characteristics of these ligand-binding sites is essential for the rational design of therapeutic molecules with improved potency, selectivity and efficacy to treat diseases and disorders associated with TRP channel dysfunction. One of the main challenges in designing *in silico* modulator that target these sites is to achieve higher affinity than natural ligands or control ligand, which have been finely honed by evolution for tight and specific interactions with their corresponding sites. Consequently, creating modulators that can compete with these natural ligands involves innovative strategies and deep knowledge of the interaction site (Clifton and Jackson, 2016).

The identification of allosteric sites has important implications for the development of drugs that specifically target ion channels belonging to the TRP superfamily and represents another promising avenue for drug discovery and the development of innovative therapeutic interventions, as allosteric and competitive modulators act through different mechanisms (Nussinov and Tsai, 2012; Nussinov and Tsai, 2014). Allosteric drugs alter the distribution of interchangeable conformational states, resulting in increased affinity (stronger protein-ligand interactions) and specificity (selectivity for a particular protein) for the target. Binding at these sites often leads to structural changes that can influence the binding characteristics at the primary site without direct competition (Guarnera and Berezovsky, 2019, 2020; Egbert et al., 2022; Xiao et al., 2022).

However, the shortcoming of computational protocols for predicting allosteric drugs lies in their inability to find their binding sites without prior knowledge, consequently the predictive accuracy and reliability of these methods are compromised, posing a challenge in CADD of allosteric drugs (Nussinov et al., 2014). Cryptic binding sites, on the other hand, are hidden or inaccessible pockets under normal conditions, even with conventional simulations, due to the slow motions of their components (Meller and Ward, 2023). Noteworthy, advancements have been made in developing algorithms to uncover these cryptic pockets, including using Markov state models (MSM) (Konovalov et al., 2021; Cruz et al., 2022) and enhanced sampling strategies (Comitani and Gervasio, 2018) or adaptive sampling approaches (Zimmerman and Bowman, 2015), as well as a combination of these methods such as classical Molecular Dynamics simulations (MDs), MSM, and Deep Learning (DL) with the gold of detecting cryptic pockets and identifying allosteric inhibitors in a pharmacological target related to cancer (Meller and de Oliveira, 2023). In this sense, the potential of AF2 to accelerate the discovery of cryptic pockets was investigated by generating ensembles of structures. They report that AF2 successfully samples open states in six out of ten known cryptic pocket examples. Simulations based on AF2-generated structures effectively sample cryptic pocket openings, overcoming the limitations of simulations using ligand-free experimental structures. MSM constructed from AF2-seeded simulations provide a reliable free energy landscape of cryptic pocket openings, demonstrating the utility of AF2 in this area (Hart et al., 2017; Meller and Bhakat, 2023).

To enhance the success rate in the design and discovery of new modulators, strategies must be employed to address the challenges posed by the low success rates of traditional docking methods. One of these problems is an unbalanced distribution between few active and many inactive states in proteins, resulting in docking runs with starting points from mostly inactive states. Some authors suggest that the modeling of proteins in the following iterations of AF2 be intentionally biased towards active states as a solution to increase the hit rate in ensemble-based docking protocols (Nussinov et al., 2023). Another factor in the low hit rate of docking is related to poorly parameterized energy terms in the scoring function used. The SF is typically derived from physics-based force fields, empirical functions, or knowledge-based terms, but lacks proper electronic treatment of the atoms involved in the protein-ligand complex. In addition, the use of fixed dielectric charges in the docking SF contributes to an increase in both false-positive and false-negative results (Khodarahmi et al., 2015). It is important to note that the electronic component of the potential energy plays a critical role in non-bonding interactions. To improve the accuracy of predicting the binding energy of a protein-ligand complex, it is necessary to accurately calculate the charge transfer and polarization, which can be achieved using quantum mechanics (QM) methods. However, applying QM methods to large biological macromolecules is computationally demanding. As a solution, an intermediate approach called QM/MM (quantum mechanics/molecular mechanics) has been developed. In the QM/MM method, only the atoms involved in the interatomic interactions within the protein-ligand complex are handled at the QM level, while the remaining parts of the system are handled using conventional molecular mechanics (MM) calculations. This combination allows for more efficient and accurate calculations of the complex (Ryde and Söderhjelm, 2016; Kollar and Frecer, 2018).

### 6.3 The rapid expansion of chemical space: challenges and opportunities in CADD

With the advent of the Internet, the change and storage of data has become possible, leading to the development of several databases containing chemical compounds or metabolites that play a critical role in research in various fields. These databases are essential for CADD, investigating studying protein-ligand interactions, exploring controlled drug release mechanisms, developing new organic materials, and conducting data-driven studies using machine learning, among other applications.

Currently, numerous open access databases of chemical compounds or bioactive molecules are available, including PubChem (Kim et al., 2023), ChEMBL (Mendez et al., 2019), ZINC (Irwin et al., 2020), BindingDB (Gilson et al., 2016), ChEBI (Hastings et al., 2016), eMolecules (https://www.emolecules.com, consulting May 2023), DrugBank (Wishart et al., 2018), HMDB (Wishart et al., 2022), IUPHAR/BPS (Harding et al., 2022), COCONUT (Sorokina et al., 2021), Drug Repurposing Hub (Corsello et al., 2017), e-Drug3D (Douguet, 2018), Promiscuous (Gallo et al., 2021) and the Natural Products Atlas (Van Santen et al., 2022). These databases are just a few examples of the resources available for research purposes and to obtain specific information.

Furthermore, many of these databases of molecular compounds are freely downloadable, while others provide diverse access methods to facilitate the work of researchers, including the use of an Application Programming Interface (API) or the implementation of web scraping techniques (Hanwell et al., 2017). An example of this is the ChEMBL database, which provides a web interface (*ChEMBL Database*) for data retrieval, a RESTful API (REST = REpresentational State Transfer) which means that it responds to a variety of programming languages such as Java, Python, JavaScript, and works with XML (Extensible Markup Language), JSON (JavaScript Object Notation) or YAML formats (Davies et al., 2015; Nowotka et al., 2017). In addition, for Python users, there is a library called the “Python client” that provides seamless access to the ChEMBL API, simplifying data retrieval (GitHub - chembl/chembl_webresource_client: Official Python client for accessing ChEMBL API, 2023). These tools become indispensable when dealing with complex queries or filtering specific datasets (data cleaning) for subsequent in-depth analysis or training models using machine learning techniques.

**FIGURE 6:**
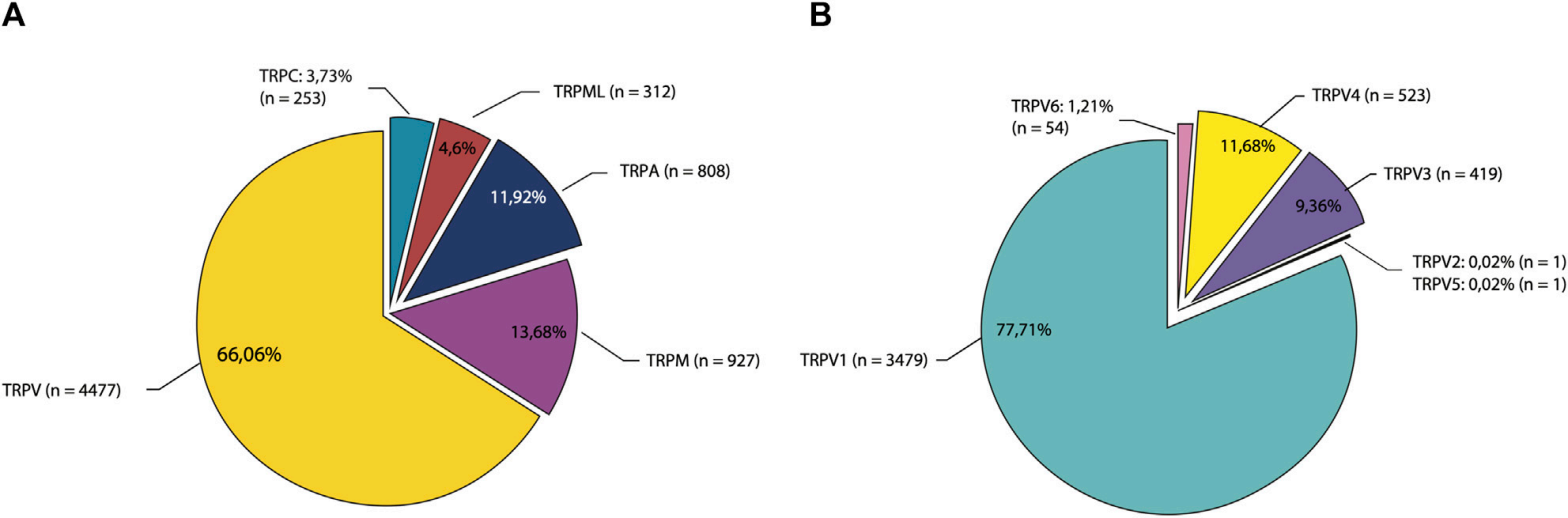
Exploring molecule dispersal rates across TRP superfamily and TRPV family members in the ChEMBL database. The pie chart shows the distribution of reported molecules in the ChEMBL database, categorized by **(A)** the entire TRP superfamily, with the TRPV family being the most represented, and **(B)** individual members within the TRPV family, with TRPV1 having the highest number of reported molecules.

For example, using a Python client, we performed a search to determine the number of reported molecules in the ChEMBL database (2023) for each member of the TRP superfamily. We specifically considered molecules reporting IC_50_ or EC_50_ values from the STANDARD_TYPE column and restricted our analysis to those reported for the TRP superfamily in humans (Papadatos et al., 2015). To date, the TRPV family accounts for 4,477 reported molecules, representing 66.06% of the total reported molecules within the TRP superfamily. The TRPM and TRPA families follow with 927 and 808 reported molecules, respectively, representing 13.68% and 11.92% of the total. The TRPML and TRPC families have the lowest number of reported molecules with 4.6% and 3.73% respectively, and the TRPP family has no molecules with reported IC_50_ or EC_50_ values (Figure 6A). Analyzing the data for TRPV family members in detail (Figure 6B), TRPV1 has the highest number of reported molecules, with 3,479, representing 77.71% of the total 4,477 molecules. The reported molecules in TRPV4 and TRPV3 represent 11.68% and 9.36% respectively, and the TRPV2/5/6 families together represent only 1.25% of the total molecules.

This significant number of molecules allows for exploratory data analysis (EDA) and the potential application of machine learning models to train predictive classifiers. This has been demonstrated in previous research, such as the development of classifiers to predict cyclooxygenase-2 (COX-2) inhibitors by categorizing molecules from the literature as highly or weakly active inhibitors (Brown et al., 2019). Additionally, Tinivella et al. (2021) described a study in which they used machine learning models to predict the activity and selectivity of inhibitors against human carbonic anhydrase (hCA), which are potential therapeutic targets. In their research, they used curated data extracted from ChEMBL, which enabled them to train their models and make accurate predictions, highlighting the value of leveraging the extensive data set to build robust predictive models.

With the rapidly expanding chemical space, researchers face the challenge of dealing with a vast number of molecules. This requires the use of efficient tools and methods. One such tool is the open-source software KNIME Analytics Platform (Berthold et al., 2009) which allows the visual creation of interconnected workflows with nodes containing custom algorithms. Within this platform, there is a GET Request node that facilitates interaction with databases that support RESTful web services, such as ChEMBL, PubChem or other biological databases. There are numerous research studies that use this tool in their workflows (Sydow et al., 2019; Kojima et al., 2020; Alegría-Arcos et al., 2022; Smajić et al., 2022), because it provides seamless integration with programming languages such as Python or R and libraries like RDkit (RDKit, 2023) and Scikit-learn (Pedregosa et al., 2012). Additionally, it facilitates integration with other commercial software programs such as the Schrödinger suite (Maestro, 2021).

In addition, Jupyter notebooks have gained significant popularity as a tool for documenting and ensuring the reproducibility of pipelines and reports in exploratory chemical data analysis and other research methodologies that use computational workflows for specific purposes. These notebooks are often hosted in repositories such as GitHub, Zenodo (Zenodo, 2023), Bitbucket (Git solution for teams using Jira, 2023), and similar platforms.

It is also worth noting that explosion of the chemical space has been driven by the need to discover new drugs, leading to the generation of combinatorial chemical libraries (Saldívar-González et al., 2020). Ruddigkeit et al. (2012) have generated a database called GDB-17 which houses more than 166 billion synthetic molecules, including various drug analogs and lead compounds for their applications in CADD techniques such as virtual screening. CHIPMUNK is another database of 95 million virtual small molecules that can be synthesized for subsequent analysis (Humbeck et al., 2018). These chemical libraries were generated using the combinatorial enumeration approach. In the enumeration approach, chemical structures are systemically generated by exploring various combinations and modifications of molecular fragments within a given scaffold. This approach allows the exploration of a vast chemical space, enabling the identification of diverse compound libraries for screening and lead optimization. In a recent study applying the enumeration approach to capsaicin, resulted in the generation of 2174 analogs. Using this approach, it was discovered four highly selective modulators of TRPV1, that exhibited greater selectivity compared to other members of the TRPV ion channel family (Bustos et al., 2022). Bioisosteres, on the other hand, involve the strategic replacement of one group or atom within a molecule with another group or atom that has similar physical or chemical properties. Bioisosteres aim to optimize the pharmacological properties of a compound while preserving its overall molecular framework. It was found a TRPV1 inhibitor through a bioisosteres protocol in the Lys-Trp (Nps) peptide scaffold (Zaccaro et al., 2011). Both techniques provide valuable tools for drug developers to enhance the potency, selectivity, pharmacokinetic properties, and overall drug-like characteristics of potential modulators for TRPV1 channels. They contribute to the generation of novel compounds with improved activity profiles and increased chances of successful drug development.

On another note, the growth of chemical space has expanded the capability to synthesize and select from a larger number of compounds than was previously possible. This has increased the synthetic feasibility of molecules or compounds in recent years, with suppliers and libraries offering readily available or custom-made molecules. These advances facilitate the acceleration of scientific research.

## 7 Conclusion

Although the TRPV1 channel has been extensively studied and its molecular intricacies are known, the discovery of specific and efficient drugs that target the channel in pathological conditions remains a challenge. Similarly, the existence of molecular elements for temperature sensing is a matter of debate in the field of ion channels. While capsaicin analogs are well suited to relieve peripheral pain when administered topically, they can cause unpleasant pain amplification in pathological pain conditions such as inflammatory or neuropathic pain. Furthermore, central administration of capsaicin or its analogs induces thermosensory and thermoregulatory distortions. In addition, although several published structures elucidate many aspects of channel function, they provide information on static states, thus limiting knowledge of important aspects of the dynamics of TRPV1 channel gating by different types of ligands. In silico tools and predictive algorithms can aid in the challenge of finding drugs that can be experimentally tested and have no unwanted side effects for the treatment of various pain and other TRPV1-related disorders.
